# Contrast Agent Design to Deliver Improved Ultrasound Echogenicity from Medical Devices: A Route to In-Ward Device Placement Tracking

**DOI:** 10.1021/acs.biomac.6c00731

**Published:** 2026-07-31

**Authors:** Amy Stimpson, Jerry Contreras, Bryce Roper, James W. Caruana, Ifty Ahmed, Cordula Hege, Joseph Sefton, Abby R. Whittington, Derek J. Irvine

**Affiliations:** † Centre for Additive Manufacturing, Faculty of Engineering, 6123 University of Nottingham, University Park, Nottingham NG7 2RD, U.K.; ‡ Department of Chemical and Environmental Engineering, Faculty of Engineering, University of Nottingham, University Park, Nottingham NG7 2RD, U.K.; § Department of Materials Science and Engineering, 1757 Virginia Tech, Blacksburg, Virginia 24061, United States; ∥ Advanced Materials Research Group, Faculty of Engineering, University of Nottingham, Nottingham NG7 2RD, U.K.; ⊥ Department of Chemical Engineering, Virginia Tech, Blacksburg, Virginia 24061, United States

## Abstract

This work reports a scalable strategy for producing polymeric medical devices with enhanced ultrasound (US) echogenicity via tailored dispersant design to support safer image-guided medical device placement. Real-time US imaging offers an alternative to X-ray imaging, reducing radiation exposure while enabling accurate device positioning/monitoring. Previous studies showed that including bioabsorbable phosphate-based glass (PBG) microspheres into thermoplastic polyurethane (TPU) could improve echogenicity. However, aggregation and processing defects limited the performance. To address this, two classes of acid-functionalized dispersants were synthesized to improve the microsphere dispersion and compatibility with TPU. These, i.e., dodecenylsuccinic diacid (DDSD) and oligomeric methacrylate/acrylates prepared using acid-functionalized chain-transfer agents, were grafted onto PBG microspheres via dip-coating and thermal treatment. Successful surface modification was confirmed by separation testing, SEM, and XPS. TPU composites containing the coated microspheres were then extruded into catheter-like structures and shown to demonstrate enhanced, more uniform echogenicity under clinical US imaging conditions without compromising the mechanical properties.

## Graphical abstract

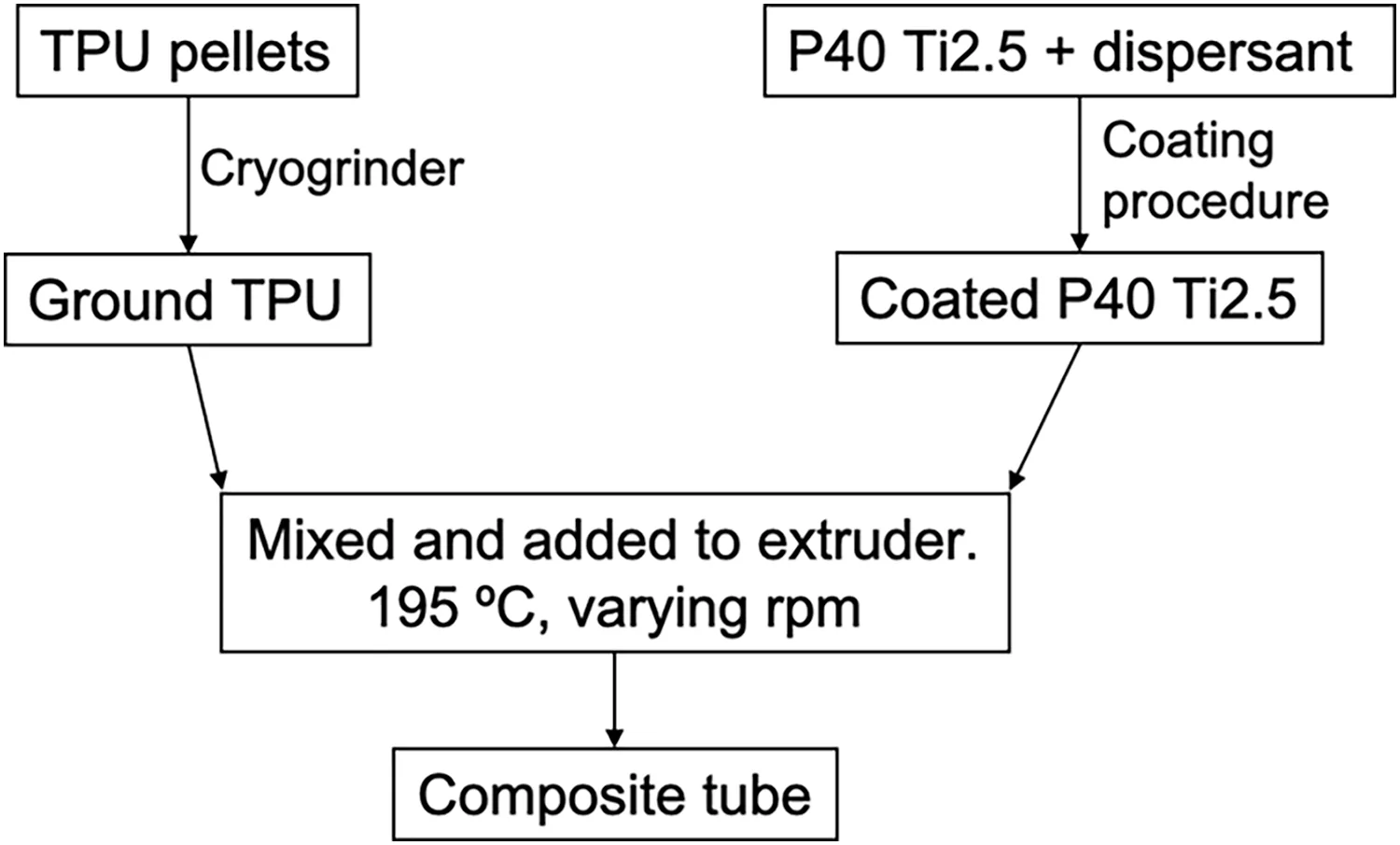

## Introduction

The use of polymer-based composite materials has become widespread within the modern polymer and biomedical industries. One definition of a polymer-based composite is a material that contains a functional “filler” material which is surrounded by a matrix polymer.^1^ The functional filler is generally present to provide a desired material property, e.g., flame retardancy, pigmentation, reduction in cost, and improved mechanical performance.^2–4^ The matrix gives the final article a definitive shape/form, protects the filler from environmental factors and can impart surface functionality/stability. The filler materials can possess many different physical forms, e.g., powders (flame retardants), fibers (reinforcements), and/or nanoparticles (pigments).^5–7^ However, a key common processing problem that exists with all types of matrix/filler compositions is a need to ensure that the particles are both well dispersed and surface wetted by the matrix materials. The former ensures fillers deliver the maximum increase in performance at minimum loadings and the latter that there are no stress centers in the final structure that result from voids being created at the filler: matrix interface.^8^ One route to achieve this is via the use of dispersants which are designed to prevent agglomeration of solid particles in liquid/molten media.^9^

Typically, dispersants are interfacially active species which are used to overcome the attractive forces that exist between solid particles and ensure that they remain separated.^9^ They generally consist of a headgroup which associates with the particle surface to anchor it in place and a tail group which protrudes away from the surface into the surrounding medium to provide steric or electrostatic stabilization.^10,11^ The former delivers particle separation by the repulsion of like charges, while the latter derives from the tail groups possessing significant molecular mass to sterically prevent particle: particle surface interaction, so preventing agglomeration.^10,11^ An additional potential benefit from the steric based variants is that their tails can be designed so they are compatible with the matrix material. Thus, their presence on the filler surface can also improve the surface wetting of the particle.^11^ The choice of headgroup of a dispersant is dependent on the particle’s surface chemistry. Typically, interactions such as ionic, hydrogen or covalent bonding between the dispersant and particle are required to obtain a stable dispersion, i.e., ensuring that the dispersant is not removed from the surface during processing. For this study, the particles of interest are bioabsorbable phosphate-based glass (PBG) microspheres.^12,13^ Prior reports highlighted suitable dispersants for PBG fillers containing carboxylic acid, hydroxyl, thiol, amine, or sodium salt groups as head groups exhibited sufficiently strong anchoring to the particle surface.^14–16^

Many synthetic routes used to produce dispersant-coated fillers rely on batch processes, such as mechanochemical, combustion, sol–gel, solid-state, micelle templating, and precipitation techniques, with the latter being the most prevalent in practical applications.^15,17–19^ A notable challenge with batch processes is that addition of a capping agent too early can also alter the morphology of the isolated particles.^18^ Consequently, many synthetic approaches now incorporate surface modifications through additional postsynthesis treatments to address this issue. For example, dodecenylsuccinic anhydride (DDSA) was successfully applied to hydroxyapatite fillers via a multistep coating procedure.^18^ In this case, the headgroup had to undergo a ring-opening stage to form the necessary carboxylic acid functionality prior to its attachment to the particles’ surface. The fact that the headgroup is initially passive was linked to both good compatibility of the dispersant with the other process reagents and the mitigation of any morphology change in the particles. Furthermore, this passive headgroup also allowed it to be applied via a continuous flow process to manufacture filler particles.^18^ This maximizes the product sustainability by reducing the number of process steps involved, minimizing waste generation and so improving the atom efficiency.

Meanwhile, in the field of clinical care, patients with peripherally inserted central catheters (PICCs) are often discharged with the catheters in place, which means that they can experience complications, such as undetected thrombosis or accidental catheter dislodgment. Typically, tracking these issues, as well as the initial device placement, is conducted via limited amounts of X-ray imaging. The amount of imaging that can be conducted is kept to a minimum to prevent negative health implications from excessive exposure to X-ray radiation. However, limiting the imaging can lead to therapeutic issues being missed. Thus, developing catheters with the capability to be tracked without X-ray imaging would greatly benefit these patients by decreasing patient stress, reducing time to diagnosis, and increasing nursing home capabilities to conduct medical device assessments, all by removing the need for an X-ray facility.

The ability to conduct real-time ultrasound (US) imaging of polymeric medical devices has been demonstrated, through the incorporation of bioabsorbable PBG microspheres into a device. The addition of the filler has been shown to produce bulk echogenic effects.^20^ The development of such echogenic catheters will potentially allow: (a) lower X-ray doses to be delivered to vulnerable patients during the initial placement, by reducing the need for multiple exposures to a single, final confirmatory X-ray procedure, (b) “in-ward” placement and position monitoring to be conducted by lower power portable US apparatus, so removing the need to transport patients to an X-ray facility, and (c) monitoring in this way will not require use of an X-ray suite nor highly trained radiographers. All of these factors will benefit the health and comfort of the patient and reduce waiting times for procedures and clinical bottlenecks.

However, in this prior work, at all filler loadings, the catheter devices exhibited levels of particle aggregation and processing defects/artifacts that were visible under US. This confirmed the need to include a dispersant for improved, homogeneous properties. Furthermore, creating these polymer-based composites was reported to increase the polymer’s echogenic properties, without undermining its mechanical and biocompatible properties.^20^ Therefore, this study attempted to deliver an improvement in the quality of US imaging to allow real-time US tracking of polymeric medical device positioning, by utilizing dispersants to improve the dispersion and integration of echogenic fillers into the polymer matrix of a catheter. One of the dispersants utilized was DDSA that had been successfully used previously,^14,16^ and its performance was compared to a series of oligomeric dispersants synthesized using thiol chain-transfer (TCT) polymerization.

## Experimental Methods

### Materials

Dodecenylsuccinic anhydride (DDSA) (mixture of isomers, technical grade, Sigma-Aldrich, 90%), methyl methacrylate (MMA, ProSciTech, 99%), ethylene glycol dicyclopentenyl acrylate (EGDPEA, Sigma-Aldrich), ethylene glycol dicyclopentenyl methacrylate (EDGPEMA, Sigma-Aldrich), di­(ethylene glycol) methacrylate methyl ether (DEGMA, Sigma-Aldrich), poly­(ethylene glycol) methacrylate methyl ether (PEGMA, average *M*_n_ = 360 g mol^–1^, Sigma-Aldrich), 3-mercaptopropionoic acid (3MPA, Acros Organics, 99%), mercaptosuccinic acid (MSA, Aldrich, 97%), 2,2-azobis­(isobutyronitrile) (AIBN, Sigma-Aldrich, 98%), cyclohexanone (Acros Organics), hexane (Fisher Scientific), dichloromethane (Sigma-Aldrich), methanol (Fisher Scientific), chloroform (Sigma-Aldrich), deuterated chloroform (CDCl_3_, Sigma-Aldrich), and tetrahydrofuran (THF, Fisher Scientific, HPLC grade) were purchased and used without further purification. Ethanol and deionized water were obtained from the University of Nottingham Chemistry stores. Texin RxT90A polyurethane, a bioinert thermoplastic (TPU) with softening point of 180 °C and degradation point approximately 283 °C, was purchased from Covestro (Pittsburgh, PA), and used as the preliminary matrix polymer.^21^ Phosphate-based glass microspheres, with a chemical composition 40P_2_O_5_–24MgO–16CaO–17.5Na_2_O–2.5TiO_2_ (referred to as P40Ti MS), were prepared via a flame spheroidization process as previously described.^13^ Gelatin 175 Bloom was provided as a gift from Vyse Gelatin Company (Schiller Park, IL), while psyllium husk (Proctor & Gamble, Cincinnati, OH) and latex Penrose drains were purchased from Amazon.

### General Synthetic Procedures

#### Dodecenylsuccinic Diacid Dispersant Synthesis via Ring Opening of Corresponding Anhydride

Deionized water (0.5 mL, 27.78 mmol) was added to dodecenylsuccinic anhydride (DDSA) (5 g, 18.77 mmol) which was contained in a flask equipped with a stirrer. The solution was refluxed overnight with agitation, after which the reaction mixture was cooled to room temperature. The product, now termed dodecenylsuccinic diacid (DDSD), was obtained in the form of a viscous pale-yellow liquid. After drying *in vacuo* at 50 °C to remove residual water, this material was subjected to characterization by ^1^H NMR. The yield was gravimetrically determined to be 4.9 g (98%).

#### Synthesis of Oligomeric Precursors and Dispersants

Dispersant synthesis was conducted using standard Schlenk line techniques following the general procedures detailed below.

#### Synthesis of Oligomeric Dispersants via the Use of Thiols as CTA

3MPA and MSA were chosen as chain-transfer agents (CTAs) for the oligomerization of MMA, DEGMA, PEGMA, and EGDPE­(M)­A. The required quantities of reagents (monomer, 3MPA, AIBN and cyclohexanone, see Table 1 for quantities) were added to a reaction vessel under an inert atmosphere and degassed under argon for 30 min. The vessel was then submerged into a preheated oil bath at 80 °C for the duration of the reaction. After the appropriate reaction time, the reactions were quenched by exposing the contents to oxygen and cooling over ice. To determine conversion of monomer to polymer product, ^1^H NMR analysis was conducted on the crude reaction mixture before purification. The product was isolated by precipitation into the appropriate antisolvent determined by the physical properties of the specific oligomer, collected via filtration, and dried in a vacuum oven (25 °C) for 24 h. GPC and ^1^H NMR analysis was performed on pure products.

**Table 1:** Polymer Data from the Polymerization of MMA with Thiol CTAs^a^

**entry**	**thiol type**	**thiol** **(mol equiv)**	***M***_**n**_ ^b^ **(g mol** ^–**1**^ **)**	***Đ*** ^a^	**EDp**	**conv** ^c^ **(%)**
1	3MPA	1.2	4500	1.3	46	90
2	3MPA	2.3	1200	1.4	11	91
3	3MPA	3.5	1100	1.3	10	85
4	3MPA	4.6	1200	1.4	11	95
5	3MPA	5.8	1400	1.3	14	93
6	MSA	1.2	400	6.4	2	95
7	MSA	2.3	700	4.2	5	96
8	MSA	3.5	600	3.7	4	95

a Reaction conditions: MMA (2 mL, 1.89 mmol), cyclohexanone (2 mL), AIBN (0.5 wt % wrt monomer), temperature = 80 °C, time = 5 h, EDp = Estimated Degree of polymerization.

b Determined by GPC.

c Determined by ^1^H NMR.

#### Preparation of DDSD Dispersant-Coated Glass Microspheres for Catheter Manufacture

A coating solution was prepared by dissolving DDSD (1.27 g, 43.00 mmol) in ethanol (50 mL). P40Ti MS (1.5 g) were added to this solution and stirred for 1 h, after which the MS were isolated by filtration and dried at 120 °C for 2 h in an oven. The dispersant-coated P40Ti MS were subsequently referred to as P40-DDSD, and the presence of the coating was confirmed by SEM and XPS analysis.

#### Preparation of Oligomeric Dispersant-Coated Glass Microspheres for Catheter Manufacture

The oligomeric dispersants were used to coat P40Ti MS following the method described here, which is detailed for MMA_2_-3MPA as an example. MMA_2_-3MPA (1.2 g, 3.92 mmol) was dissolved in cyclohexanone (50 mL). To this solution, P40Ti MSs (1.5 g) were added, and the solution was stirred for 1 h, after which they were collected by filtration and dried at 120 °C for 2 h. The isolated microspheres were added to cyclohexanone (25 mL), which was stirred for 30 min to remove any further unattached dispersant. They were then collected by filtration and dried at 120 °C for 2 h. The samples were then subjected to the biphasic separation test, described below, to confirm that the surface chemistry had been altered because of the coating process. The dispersant-coated PBG microspheres are subsequently termed P40-(MMA_2_-3MPA).

### General Characterization Techniques

#### Nuclear Magnetic Resonance (NMR)

^1^H NMR was performed on a Bruker DPX-300 spectrometer (300 MHz) at 25 °C. Samples were prepared as 10 mg/mL solutions in deuterated chloroform (CDCl_3_) or deuterated dimethyl sulfoxide (DMSO-*d*_6_) to which chemical shifts, measured in δ_H_ (ppm), were referenced to (residual CHCl_3_ at 7.26 ppm and residual DMSO at 2.50 ppm). Analysis of spectra was carried out using Mestrelab Mnova 10.0 software.

#### X-ray Photoelectron Spectroscopy (XPS)

XPS spectra were acquired using a Kratos Axis Ultra DLD spectrometer with a monochromated Al Kα X-ray source (hν = 1486.6 eV), hybrid electron optics, a hemispherical analyzer, and a Delay Line Detector (DLD) at a 30° collection angle and a 90° takeoff angle. The X-ray gun power varied between 100 and 150 W, depending on the vacuum quality. Spectra were recorded with a 300 × 700 μm^2^ aperture, using an 80 eV pass energy for survey scans and 20 eV for high-resolution scans. Data acquisition employed Kratos VISION II software, with files converted to VAMAS format. Samples were grounded, and no charge compensation or correction was applied; energy calibration utilized Au 4f 7/2, Ag 3d 5/2, and Cu 2p 3/2 peaks. Samples were prepared on double-sided sticky tape and the analytical chamber’s base pressure was maintained at 3 × 10^–9^ Torr. Data processing was carried out using CasaXPS software (version 2.3.25).

#### Gel Permeation Chromatography (GPC)

GPC was performed on an Agilent 1260 Infinity system (Agilent Technologies, USA) equipped with a PL-gel 5 μm guard column and two PL-gel mixed-D (7.5 mm × 300 mm) columns in series. THF was the mobile phase at a flow rate of 1 mL/min. A Wyatt Optilab light scattering detector and a differential refractometer (DRI) were used for sample detection and analysis. The differential index of refraction value (d*n*/d*c*), 0.0816, was used to calculate polymer molecular weight and dispersity. GPC samples were made up as 2 mg/mL solutions in HPLC grade THF which were filtered into a sample vial through a syringe filter (Whatman, 25 mm, 0.2 μm). Analysis of the spectra obtained was carried out using Astra software.

#### Thermogravimetric Analysis (TGA)

TGA was performed on a TA Instrument Discovery. Samples were analyzed in high temperature platinum pans in an N_2_ environment, with a heating rate of 10 °C/min from ambient temperature up to the desired maximum temperature. Analysis was carried out using TA Instruments TRIOS software.

#### Scanning Electron Microscopy (SEM)

SEM was performed to determine the P40Ti MS size. Particles were mounted on an SEM stub using carbon tape and coated using a platinum coater (120 s, 2.2 V, Polaron SC7640) to provide a conductive surface and prevent charge buildup. Particles were imaged using a Phillips XL30 SEM at varying magnifications. The sample average particle size was determined by the average diameter of 100 particles using ImageJ software. Extruded materials with and without microspheres were sectioned into 1 cm lengths and then freeze-fractured in liquid nitrogen to avoid breaking of particles. The sections were allowed to warm to room temperature before being attached to carbon tape and sputter-coated with 7 nm iridium. An FEI Quanta 600 environmental SEM with backscatter imaging allowed for analysis of particle size, composition, and visualization of distribution in the polyurethane, while secondary emission imaging revealed surface morphology of samples. A solid-state backscatter electron detector was used to visualize the embedded microspheres within the TPU matrix.

#### Biphasic Separation Test to Demonstrate Surface Functionality

This test was designed to confirm that attachment of the dispersant to microspheres had created a change in the surface properties of the spheres. The hypothesis behind the test was that coated and uncoated microspheres will have a propensity to reside in either the organic or aqueous phase of a biphasic system of oil and water (Figure 1), dependent on whether their surface is hydrophilic or hydrophobic.

**Figure 1:**
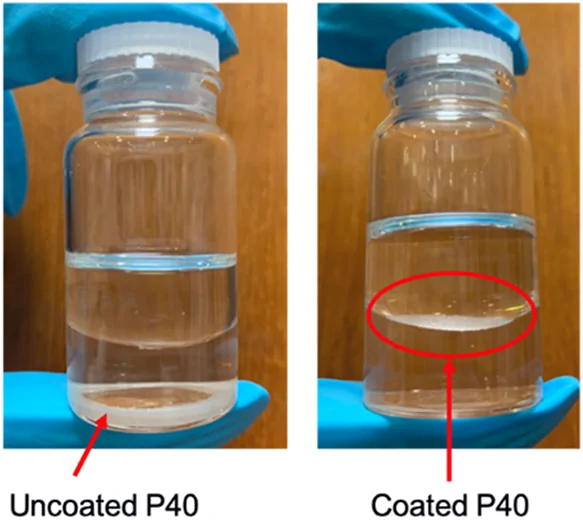
Images of the biphasic suspension test that demonstrates the coated spheres are retained in the upper (oil) layer while the uncoated PBG spheres progress through the interface and are concentrated in the bottom (water) layer.

In this study, the uncoated microsphere surface is hydrophilic, while the coated versions will exhibit a hydrophobic exterior. Thus, the biphasic suspension test was devised by utilizing the following procedure. To a vial containing 2 mL of toluene and 2 mL of deionized water was added approximately 10 mg of microspheres. The uncoated microspheres (hydrophilic) were observed to drop and settle immediately at the bottom of the water phase as seen on the left image (i.e., the bottom of the vial). However, the dispersant-coated microspheres (hydrophobic) were noted to settle at the interface between the organic and aqueous layers, forming a thin layer (Figure 1). This indicated a change in their surface chemistry upon coating. Additionally, a control group of microspheres, i.e., a batch that had been subjected to the coating procedure in the absence of dispersant, was also tested. The control group was found to behave as uncoated microspheres did and collected at the bottom of the aqueous phase, indicating that no change in surface chemistry had occurred in the absence of the dispersant.

#### Catheter Fabrication

A Haake Minilab Microcompounder (Thermo Fisher Scientific, Waltham, MA) with a custom annular die was used to extrude hollow tubes comprising dispersant-coated microspheres and TPU. A total of 5 g (ground TPU pellets and ≤5 wt % dispersant-coated microspheres) were added to the hopper and manually pushed into the cavity. The extruder was preheated to 195 °C and the speed of extrusion was varied.

#### Soft Tissue Phantom Fabrication

Soft tissue phantoms were fabricated the day before US sessions, as previously reported using latex tubing as venous mimics.^20,22^ Extruded samples were placed inside the tubing, deionized (dI) water was added, with any bubbles being removed before tubing was tied. Fluorocarbon fishing line was attached to the knotted ends of the venous mimics and pulled taut, allowing for samples to be held in place inside a rectangular mold. Psyllium husk was sieved through a 212 μm filter and lightly ground with a pestle and mortar, to remove any clumps of powder. A 10 wt % gelatin with 5 wt % psyllium solution in preheated water at 60 °C, and the solution filtered through wire mesh to remove clumping. The mixture was poured into the rectangular mold around the vein mimics. The filled mold was covered with parafilm and placed inside a 4 °C refrigerator to set overnight. After refrigeration, samples would be located between 2 to 3 cm from the surface of the tissue phantom.

#### Echogenic Properties

The prepared tissue phantoms were imaged under US using a Philips IU22 ultrasound with a 12 MHz linear transducer. The location of the samples was first imaged in the transverse direction to confirm the relative depth and position of the vein mimetics. Then, images were captured in the longitudinal direction in the order of placement in the phantom. The gain was kept constant between imaging sessions to offer ease of comparison of the signal-to-noise ratios (SNRs) across all samples.

The US images were processed by using Andor Solis I image processing software. Regions of interest were selected in the bulk mimetic above and below the vein mimetic to analyze shadowing and SNR of the mimetic to test for consistency between mimetics. Six regions 10 pixels in height and 64 pixels in width were selected from the lumen of the tube section for processing in Andor Solis I, and the SNR was calculated. The resulting SNR of each sample was compared with the unfilled TPU control to determine the percent change in SNR created by the presence of the microspheres.

#### Statistical Analysis

ANOVA (analysis of variance) was performed between loading levels and between compatibilizers to test for statistical significance of the loading and of the dispersants being used on the SNR. Data are reported as the average ± standard deviation.

### Semiquantitative Image Analysis

SEM images were analyzed to quantify microsphere dispersion using a semiquantitative Python-based workflow. A dark-feature area fraction was calculated as the ratio of dark pixels to total pixels. Spatial heterogeneity was quantified by dividing each image into a 10 × 10 grid, calculating local intensity variance within each region, and computing the variance of these values to obtain a spatial variance index. Metrics were averaged across multiple images for each condition to compare dispersion and clustering behavior.

## Results and Discussion

In this study, surface functionalization of the dispersant-coated microspheres was achieved by utilizing a simple wet chemical grafting technique. The treatment method introduced the glass spheres to the dispersants that contained functional head groups that would create associative interactions to the microsphere surfaces, enhancing their compatibility with polymeric dispersants. Following the solution-based mixing, to facilitate the dispersant coupling, the microspheres were collected by filtration and dried at 120 °C. This latter stage was conducted at higher temperature, which was used to promote the formation of covalently attached functional groups to the microsphere surfaces. This combined approach of solution mixing followed by the application of heat was adopted to promote stable dispersant attachment, improved dispersion in the TPU matrix, and an enhanced US echogenicity.

This bonding optimization is important because the coating is deposited onto the surface of the filler of a polymer-based composite, and a potential issue is its ability to be retained on the surface of the filler during postprocessing. Demonstrating this is quite difficult because the level of coating is very small; therefore, it is difficult to achieve an analytical method to accurately detect/measure any leach material. Furthermore, attempting to extract filler from the composite leads to high levels of damage; therefore, the loss of coating could not be determined. However, the authors are confident that the coating will be retained over the operational lifetime of the medical device. This conclusion was based on their study of the interfacial shear strength of PLA composites in which end-functional oligomer-coated glass fibers were included.^14,23^ Subsequent hydrolytic degradation of a composite showed that the presence of the coating after processing was dependent on the suitability of the headgroup for the filler surface. With an appropriate headgroup oligomer in place, the fibers (a) could not be removed from the matrix as the bond between fiber and matrix was still in place and (b) the coating protected the fiber from being hydrolytically damaged itself. This suggested that the coating was well adhered and would not degrade while in use. Therefore, as the headgroup has been optimized for this application, the authors believe the coating will behave in a similar fashion.

### Strategy for the Design of Dispersants for Contrast Agent Preparation

Previous research by the authors had demonstrated that the addition of dispersants and/or compatibilizing agents to PBG fibers could produce both an improved fiber dispersion and better fiber:matrix polymer material interfacial bonding.^24,25^ Thus, the coating strategy chosen in this study combined the learning from both of these prior programs, to focus on materials containing an acid headgroup the initial dispersion/compatibilization of the P40Ti (i.e., the PBG) microspheres (MS). These species were the (a) diacid headgroup dodecenylsuccinic diacid (DDSD), which was readily obtainable via the ring opening of the commercially available dodecenylsuccinic anhydride, and which contained a hydrocarbyl tail-group and (b) oligomeric species that controlled by acid-functional thiols of monomers with pendant group structures that may have greater affinity with the soft block of the TPU matrix synthesized using thiol chain transfer (TCT). The monomers from which the oligomeric dispersants were synthesized were methyl methacrylate (MMA), diethylene glycol methacrylate (DEGMA), polyethylene glycol with *M*_n_ = 360 (PEGMA_360_), ethylene glycol dicyclopentenyl ether methacrylate (EGDPEMA), and ethylene glycol dicyclopentenyl ether acrylate (EGDPEA) (see Figure 2).

**Figure 2:**
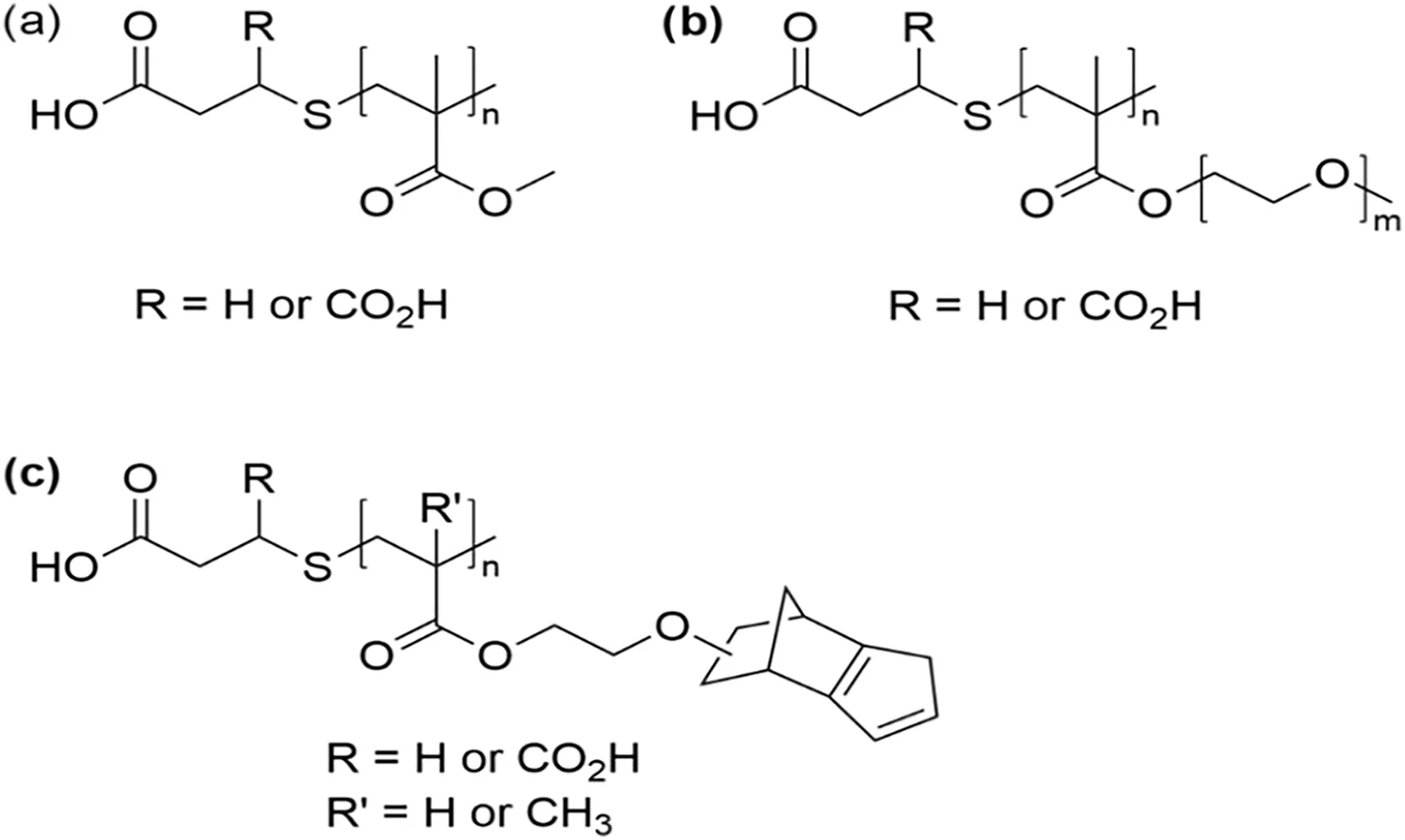
Molecular structures of target oligomeric species when controlled with 3MPA (R = H) or MSA (R = COOH) as the thiol chain-transfer agent: (a) MMA, (b) If *m* = 2 – DEGMA, if *m* = 8 – PEGMA_360_, and (c) if R′ = H – EGDPEA, if R′ = CH_3_ – EGDPEMA.

TCT is particularly attractive because thiols serve simultaneously as chain-transfer agents (CTA) and functional head groups capable of surface anchoring. Meanwhile the oligomeric tails can be tuned to match the polarity, hydrophobicity and segmental chemistry of the TPU matrix. Additionally, chain-transfer with thiols can be achieved in flow to enable process intensification so as to reduce process/economic footprint.^26^ In this respect, TCT is more favorable to adopt when compared to the well-documented catalytic chain-transfer (CCTP) method of making functional oligomers,^27,28^ This is because CCTP needs a two-step process to make the acid-functional dispersant. The control chemistry leaves a disproportionation double bond terminal group, so to complete the synthesis of the dispersant, this bond must be used as a center for further reaction, such as a β-scission mechanism with a radical,^27–29^ in combination with another controlled polymerization agent,^30–32^ being subjected to thermal treatment,^33^ or via the use of thiol–ene click chemistry.^34,35^ Furthermore, thiols have long been recognized as highly efficient CTAs in free-radical polymerization. Recent work has shifted from simply reporting chain-transfer constants to strategically exploiting thiol reactivity to engineer advanced materials, including covalent adaptable networks (CANs),^36^ liquid-crystalline elastomers (LCEs),^32^ degradable photopolymers, and additive-manufacturing resins.^37^

MMA was included as it is a well-studied model methacrylate that can be used to evaluate the performance of the control chemistry. DEGMA and PEGMA_360_ contain poly­(ethylene oxide) (PEO) pendant groups similar in structure to the soft block of the TPU, such that they should have an affinity for the soft block of the TPU groups. EGDPEA and EGDPEMA both contain a hydrophobic cyclic group in their pendant groups but also have the ethylene oxide chain, so they may also be compatible with both segments of the PU matrix. Furthermore, the acrylate has been identified as possessing microbial antiattachment properties.^38^ This means that films containing poly­(EDGPEA) have been shown to prevent the accumulation of biofilms.

The introduction of acid anchoring groups into the oligomer structure was achieved via the use of the acid-functionalized thiol chain-transfer agents (CTA). In this study, adhesion of the dispersant to the P40Ti MS was achieved by solution-based dip-coating rather than adopting the elevated-temperature hydrothermal route to prevent any degradation of the oligomers due to exposure to elevated temperatures.

### Dispersant Synthesis

The DDSA ring-opening reaction was demonstrated to be efficient and used mild reaction conditions, i.e., water reflux. No solvent was used, so no purification of the products was required other than drying, see Figure 3.

**Figure 3:**
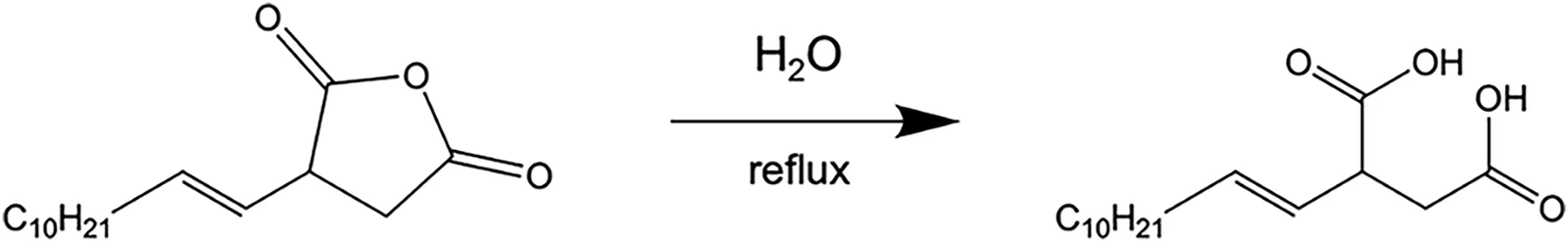
Synthetic reaction for ring opening of DDSA with water.

Meanwhile, the oligomeric dispersants were synthesized by utilizing thiol chain-transfer agents (CTA) to control the polymerization reaction. Thus, simply by changing the thiol CTA, the oligomer end functionality could be changed. Two thiols, 3-mercaptopropionic acid (3MPA) and mercaptosuccinic acid (MSA), that both contained carboxylic acid moieties were used to introduce suitable head-groups for attachment of the proposed dispersants to the P40Ti MS contrast agents.^14,18^ (see Figure 4).

**Figure 4:**
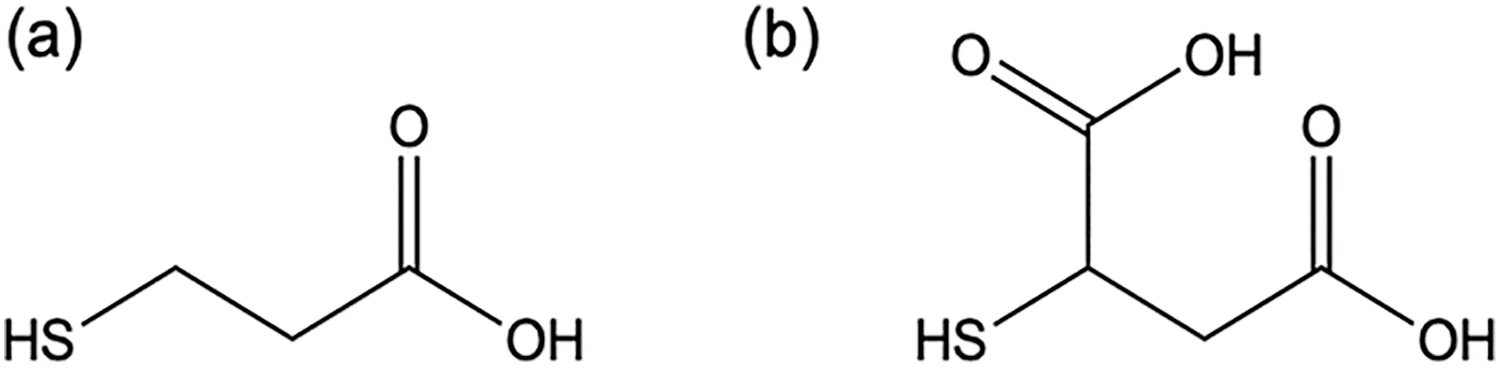
Molecular structures of thiol CTAs. (a) 3-Mercaptopropionoic acid (3MPA) and (b) mercaptosuccinic acid (MSA).

Thus, a series of model MMA reactions were conducted to find optimal conditions for the use of 3MPA and MSA as CTAs to produce these oligomeric dispersants (Table 1).

3MPA performed well as a CTA under the conditions described, with the *Đ* values obtained between 1.3–1.4 and conversions above 90%. However, the Mn values for entries 2, 3, 4, and 5 were all relatively similar, i.e., they ranged between 1100 and 1400 g mol^–1^, suggesting that the limiting concentration of this specific thiol is ∼2.3 mol equivalents (Table 1, Entry 2). Meanwhile, MSA (Table 1, Entry 6–8) was shown to be less efficient as a CTA than 3MPA, i.e., lower Mn values were recorded but more polydisperse materials had been produced. This was attributed to the fact that MSA is a solid, which was insoluble in the MMA monomer, and only partially soluble in the cyclohexanone solvent due to the high concentrations used in these experiments. Additionally, as these materials have a diacid headgroup, it was proposed that there may be an additional interaction with the GPC column gel that may result in broad *Đ* values recorded. Thus, it was proposed that these materials may still have utility as dispersants based on this potential increased surface interaction due to the second acid functionality.

The model MMA experiments had shown that both thiols could produce oligomers, so they were applied to the synthesis of the ethylene glycol-containing oligomers of interest for this study (see Figure 4). These polymerization results are displayed in Table 2, and reactions were initially optimized using the cobalt-catalyzed CCTP method, see Table 1, where the estimated degree of polymerization (EDp, i.e., GPC Mn value minus the headgroup weight divided by repeat unit formula weight) is provided to enable a better comparison between the oligomers with varying repeat unit sizes.

**Table 2:** Oligomeric Dispersants Produced via the Thiol-Controlled Reactions of Ethylene Glycol Macromonomers with Thiol CTAs^a^

	**monomer**	***M* (mL)**	***S* (mL)**	**thiol type**	**thiol (mol equiv)**	***M***_**n**_ ^b^ **(g mol** ^–**1**^ **)**	***Đ*** ^b^	**EDp**	**conv** ^c^ **(%)**
1	DEGMA	2	2	3MPA	1.2	3000	1.4	15	96
2	DEGMA	2	2	3MPA	2.3	1600	1.4	8	95
3	DEGMA	2	2	3MPA	3.5	1300	1.4	6	97
4	DEGMA	2	2	3MPA	4.6	1100	1.3	5	96
5	DEGMA	2	2	3MPA	5.8	1000	1.3	5	95
6	DEGMA	2	2	MSA	1.2	4000	1.5	20	97
7	DEGMA	2	2	MSA	2.3	2700	1.3	14	95
8	DEGMA	2	2	MSA	3.5	2200	1.2	11	96
9	PEG_360_MA	6	12	3MPA	0.75	800	1.7	2	93
10	PEG_360_MA	6	12	3MPA	0.4	1500	1.9	4	96
11	PEG_360_MA	6	12	MSA	0.56	2600	1.4	7	96
12	PEG_360_MA	6	12	MSA	0.75	1800	1.4	5	95
13	EGDPEA	2	4	3MPA	0.13	3000	1.5	12	85
14	EGDPEA	2	4	3MPA	0.26	1500	1.4	6	96
15	EGDPEA	2	4	3MPA	0.53	3100	2.7	12	95
16	EGDPEMA	2	4	3MPA	0.14	3200	1.6	11	94
17	EGDPEMA	2	4	3MPA	0.28	1600	1.4	5	98
18	EGDPEMA	2	4	3MPA	0.57	1000	1.4	3	88

a Reaction conditions: Monomer = M, solvent = S = cyclohexanone, AIBN (0.5 wt % wrt monomer), temperature = 80 °C, time = 5 h. EDp = estimated degree of polymerization.

b Determined by GPC.

c Determined by ^1^H NMR.

Similar to the model MMA oligomerizations, all concentrations of 3MPA utilized in reactions with the DEGMA macromonomer produced oligomers in the 1000–3000 range (estimated DP (EDp) range 5–15) that exhibited narrow dispersity values ranged from 1.3–1.4 and greater than 95% conversion (Table 2, Entries 1–5). Meanwhile, the reactions performed with MSA (Table 2, Entries 6–8) demonstrated significantly smaller Đ values (in the region of 1.4) with DEGMA and PEGMA, when compared with the MMA reactions, due to its greater compatibility with the monomer. Their typical EDp’s ranged from 5–20 with conversions of 95–97% achieved. The PEGMA oligomers exhibited low degrees of polymerization when a 2:1 ratio of solvent: monomer ratio was used (Table 2, Entries 9–12). Both thiols performed as very effective CTAs for PEGMA. However, 3MPA still performed more efficiently at the same molar equivalent level (e.g., 0.75 mmol eq). Therefore, due to its better CTA performance, all reactions with the EGDPEA and EGDPEMA were conducted with 3MPA. It was shown to produce oligomers, delivering similar *M*_n_ and *Đ* values with both monomers, but only when conducted at a ratio of 1:2 monomer: solvent (v:v). Lower solvent ratios resulted in significant increases in viscosity during the reaction which was attributed to the onset of cross-linking.

These results demonstrated that the chosen monomers could successfully be converted into oligomers by using both thiol CTAs in high yields. Thus, the process was scaled up to 6 mL of monomer to produce enough material to allow samples of P40Ti MS to be dip-coated with a dispersant. These reactions were performed using the higher, 1:2 monomer:solvent (v:v), solvent level to ensure that increasing the scale of the reaction did not lead to a thermal runaway. Ten oligomers were synthesized at this scale and were all produced in high conversions, i.e., above 90%, see Table 3.

**Table 3:** Overview of Second-Generation Dispersants^a^

**entry**	**monomer**	**thiol**	**thiol** **(mol equiv)**	***M***_**n**_ ^b^ **(g mol** ^**–1**^ **)**	**EDp**	***Đ*** ^a^
1	MMA	3MPA	0.06	1700	16	1.5
2	EDGPEA	3MPA	0.13	2500	10	1.5
3	EDGPEMA	3MPA	0.14	2000	7	1.6
4	PEG_360_MA	3MPA	0.75	800	2	1.7
5	PEG_360_MA	3MPA	0.40	1500	4	1.9
6	MMA	MSA	0.10	3700	35	1.2
7	EDGPEA	MSA	0.19	3400	13	1.5
8	EDGPEMA	MSA	0.21	4400	15	1.6
9	PEG_360_MA	MSA	0.75	1800	5	1.4
10	PEG_360_MA	MSA	0.40	2600	7	1.4

a Reaction conditions – Monomer (6 mL), cyclohexanone (12 mL), 3MPA or MSA (mol equiv relative to monomer), AIBN (0.5 wt % wrt monomer), 80 °C, 5 h.

b Determined by GPC.

### Manufacture of the Contrast Agents—Coating Particles with Dispersants

The contrast agents were then coated onto the particles using a novel, solvent-based dip-coating method that did not expose the materials to a temperature greater than 120 °C to avoid inducing any degradation of the oligomers. This involved soaking the P40Ti MS in an ethanol solution of the crude DDSD or oligomeric dispersant solutions, which were then isolated and washed, as described in the experimental method. This dip process was designed to allow the dispersant to make associative bonds to the surface of the particle. Following this, they were collected by filtration and subjected to a drying stage at 120 °C to both drive off residual solvent and promote the potential to create formal covalent bonds between the dispersant and the surface. The dispersant-coated particles were then subjected to evaluation in the initial biphasic separation screening test. This confirmed that the solubility properties of the coated particles were different from those of their uncoated equivalents. The former was collected at the oil:water interface, while the latter was collected at the bottom of the vial within the aqueous phase. This was taken to be evidence that the dispersant had both successfully coated and remained adhered to the P40Ti MS.

The crude solution was used because it was proposed that the small amount of residual monomer present would not affect the attachment of oligomers to P40Ti MS, since the monomers did not contain the desired headgroup. To confirm this, a separate solution of the MMA monomer and thiol in cyclohexanone was prepared and used in the coating procedure. The P40Ti MS from this experiment were shown to fail the biphasic suspension test, i.e., the MS settled to the bottom of the aqueous layer as was observed for the uncoated MS. This confirmed that the purification of the crude polymerization solution was not necessary for successful coating.

The coated P40Ti MSs were also subjected to SEM and TGA analysis both pre- and postcoating. This analysis showed that no degradation of the particles had occurred during the coating procedure, see Figures S1 and S2 (SEM) and Figures S3, S4 and S5 (TGA). The SEM data showed no change in particles size nor polydispersity for the P40Ti MS resulting from dip-coating. Meanwhile, the thermograms obtained from multiple pre- and postcoating samples showed that they possessed very high thermal stability, exhibiting no significant weight loss from the samples were observed across a temperature ramp from ambient up to 600 °C. Typical total percentage weight loss values observed were 0.16 and 0.13% for the naked and coated particles respectively, which is within error of the equipment.

However, these mass loss values were below the detection limit of the TGA equipment, so did not directly confirm the presence of the dispersant. To confirm where the coating mass losses should be observed, a sample of the DDSA that had been ring opened by thermal treatment in the presence of an excess of water was subject to TGA analysis. It was found to exhibit two distinct mass loss transitions (see Figure S3). The first (onset temperature 95 °C, weight loss 10%) was attributed to water not removed during drying and/or has been associated with the carboxylic functionalities of the ring opened headgroup. Meanwhile, the second (onset 198 °C, weight loss 89%) was associated with the thermal decomposition/devolatilization of the ring opened C_12_ diacid species. Thus, as the coated P40Ti MS TGA spectra did not exhibit mass losses in these regions, these data could only confirm the thermal stability of the MS samples remained unaffected after the coating procedure. Thus, if the coating was present, the introduction of the acidic groups within its structure did not promote additional degradation at elevated temperatures.

Consequently, both uncoated and DDSD/oligomer-coated P40Ti MS were subjected to XPS analysis to investigate their surface chemistry, and these data are contained in Table 4.

**Table 4:** Elemental Composition of Uncoated, and DDSD and MMA_2_-3MPA Coated P40Ti MS Samples Determined by XPS

	**elemental composition** **(mol %)**									**normalized ratios**	
**P40MS sample**	**C**	**O**	**P**	**Ca**	**Na**	**Mg**	**Ti**	**S**	**Si**	**C:P ratio**	**O:P ratio**
theoretical	0.0	47.6	28.1	7.3	9.1	6.5	1.4	0.0	0.0		1.7
uncoated	20.6	49.3	14.8	3.6	6.4	3.9	0.0	0.0	1.4	1.4	3.3
DDSD	31.6	43.7	11.3	2.9	3.4	4.3	0.0	0.0	3.2	2.8	3.9
MMA_2_-3MPA	32.0	37.5	9.8	3.4	2.0	11.2	0.0	1.9	2.2	3.3	3.8

The elemental composition of the surface of a sample of various P40Ti MS was obtained from an average of six wide-scan spectra. In these XPS experiments, a charge neutralizer was used, which resulted in all peaks being slightly displaced. Thus, the dominant peak, which was attributed to an aliphatic carbon environment, was used as a calibration point at 285 eV binding energy to correct the binding energy scale.

Examination of the XPS data of the uncoated particles showed there was a significant amount of carbon (∼21%) present in the sample. This was attributed to processing materials that had become attached to the sample during preparation. Closer inspection of the data showed that it also influenced the oxygen percentage as well as the carbon data. The remainder key elemental composition of the P40Ti MS was as expected, comprising oxygen, phosphorus, calcium, sodium, titanium, and magnesium. Additionally, a small amount (∼1%) of silicon was detected; however, this is another typical artifact of XPS and can be introduced during sample preparation. An example wide-scan spectra of P40-DDSD with peaks assigned to elements have been included in the ESI document as Figure S6.

The coated particles had been prepared following the same methodology, so the carbon and oxygen data have been expressed as both a C:P and O:P ratio in order to normalize the relative additional carbon and oxygen content on the coated particles to define if there were measurable differences. The values of both ratios were noted to have increased in the coated particles, which was tentatively attributed to the DDSD or MMA_2_-3MPA coating being present on the surface of the particles, having displaced the process residues observed in the uncoated samples. DDSD is a molecule that solely contains carbon and oxygen, so it influences only these peaks. However, the MMA_2_-3MPA does contain a heteroatom as part of the sulfur CTA fragment, which was detected in the XPS analysis of the MMA_2_-3MPA-coated P40Ti MS, supporting the conclusion that the oligomeric coating was present. Furthermore, unlike the uncoated MS, there were clearly different carbon and oxygen environments present in the differing components visible within the high-resolution carbon scan, suggesting that differing functional groups were also present. Again, this supports the conclusion that the dispersants had been successfully deposited on the microsphere surfaces (see Figures S7 to S9).

### Design and Manufacture of Catheters Containing the P40Ti Contrast Agent Microspheres

Hollow catheter samples containing up to 5 wt % P40Ti MS either with or without coatings were all successfully fabricated using the Haake twin screw microcompounder. After the composite was processed, backscatter ESEM was performed. For the uncoated P40Ti MS, defects arising from the extrusion procedure can be seen in Figure 5, as indicated with the yellow highlighted areas.

**Figure 5:**
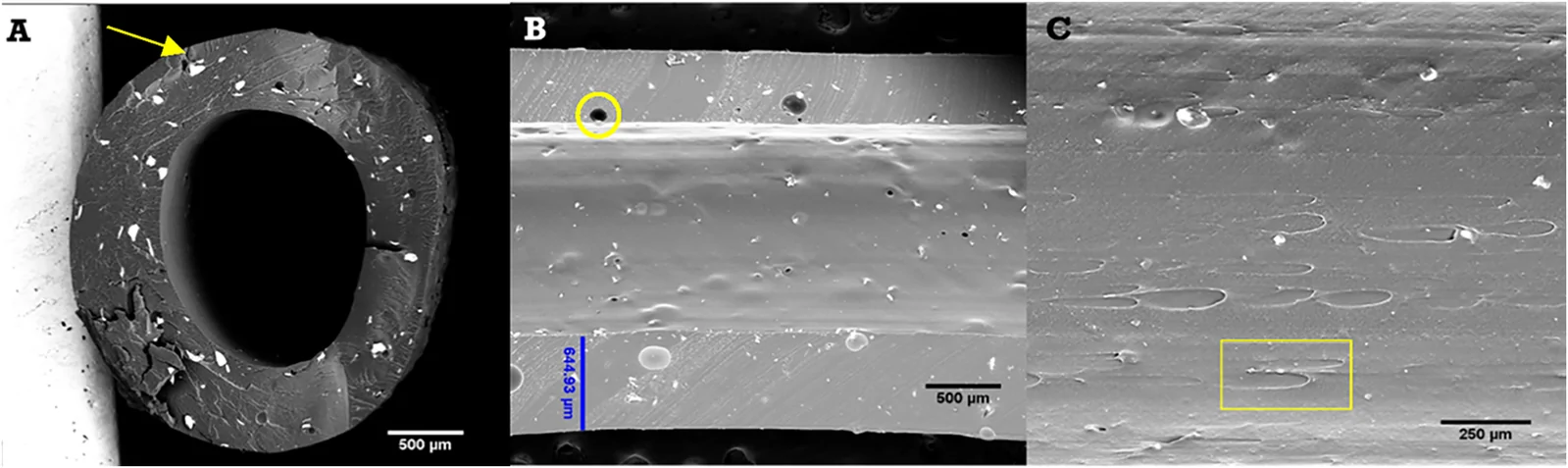
Representative backscatter ESEM images of extruded tube samples containing uncoated contrast agent microspheres, showing common processing induced defects. The cross section of tube after cryofracture with fractured particles (A, arrow) and longitudinal section with air pockets in the wall (B, circle) and shear defects (C, box). Inconsistent wall thickness was observed (A, B). Scale bars A and B = 500 um and C = 250 um.

The defects described below were particularly pertinent in samples containing uncoated glass microspheres, such it was hypothesized that their presence was increasing the difficulty in processing the composite material. All parts of Figure reveal that some glass microspheres have been crushed or fractured during processing. This reduced the density of the echogenic particles and was likely to result in a reduced US response. Figure 5A shows that the thickness of the wall was inconsistent across the tube. After exiting the extruder, the tube was still molten and could be pulled or stretched to decrease the lumen size. However, it would appear that this uncoated MS material had behaved inconsistently to this force, leading to an inconsistent, tube diameter and wall thickness and causing the defects as shown here. It was proposed that this may result from a poor level of overall dispersion of the MS in the matrix material. Figure 5B,C show air bubbles and shear marks in the polymer matrix and in the wall of the tube, respectively. These are attributed to the nature of the matrix/processing conditions allowing air bubbles to become trapped in the molten polymer. Again, this was hypothesized to be related to the presence of the uncoated MS filler, as the matrix on its own was found to process well under the same conditions applied. In this case, it was proposed that the poor wetting on the filler by the matrix may allow the formation of air voids, particularly on the surfaces of the tube, are not desirable. While such air bubbles have been used to contribute to echogenicity, both their uneven distribution and increased surface roughness are not considered advantageous in this study.^39–42^ The former would give highly inconsistent imaging performance, and the latter could also provide a potential site for bacterial biofilm formation.

By comparison, Figure 6 contains a representative SEM image of an optimal extruded sample using PEGMA oligomer dispersant-coated MS contrast agents, it shows no obvious defects or crushed particles, rather that they are intact and not agglomerated. Additionally, there should be a good dispersion of P40Ti MS throughout the tube, and the wall thickness is consistent across the tube.

**Figure 6:**
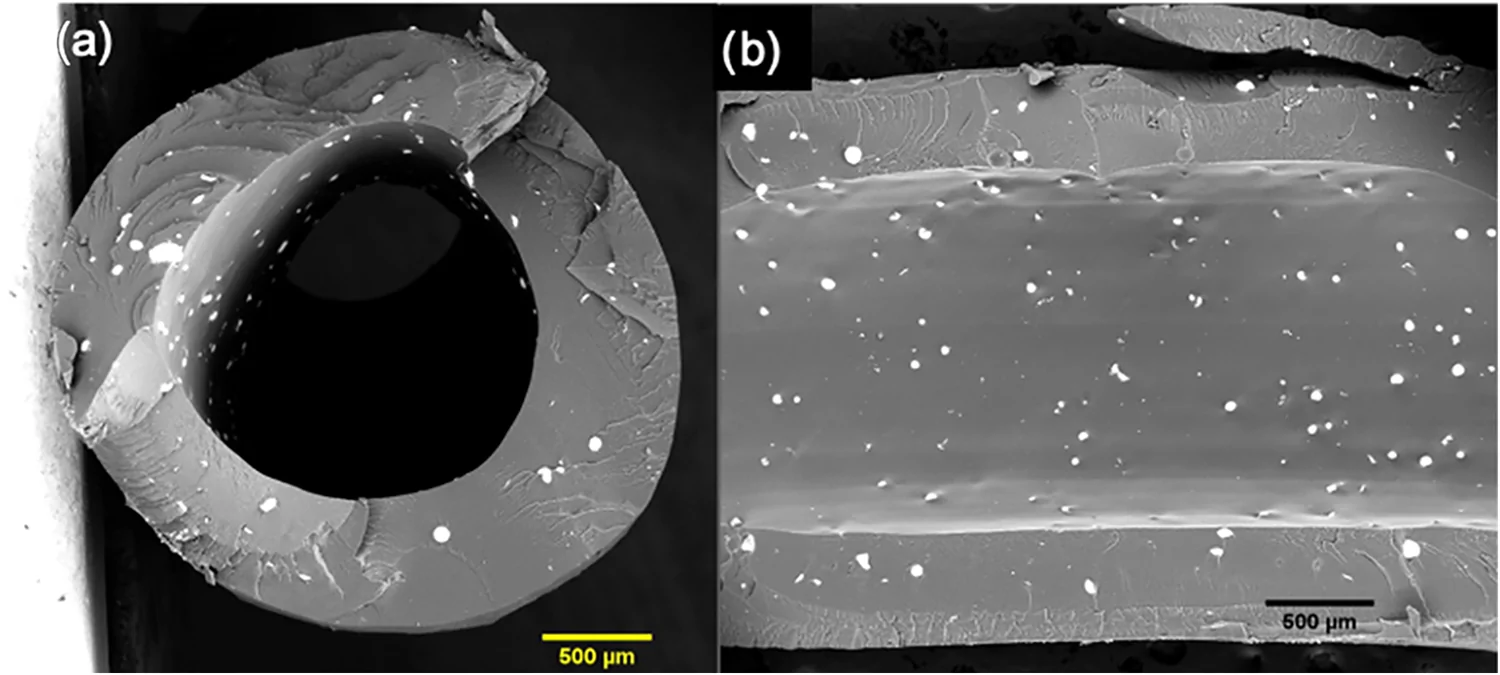
Representative backscatter ESEM images of optimal extrusion sample with coated P40Ti MS in cross section (a) and lengthwise (b) views of sample after cryofracture. Wall thickness appeared to be uniform. Both Scale bars are 500 um.

It can be seen from Figure 6 that there was little agglomeration of particles in the tube sample. The outer wall of the tube was of uniform thickness and did not appear to contain air bubbles or other defects described previously, such as shear marks.

Consequently, a semiquantitative analysis of key SEM images was performed using (i) dark-feature area fraction and (ii) a spatial variance index derived from block-wise intensity analysis in Python. This was conducted to support the visual inspection conclusions drawn about the improvement in dispersion based upon the use of a dispersant. At 1 wt % loading, both dispersant-treated and untreated samples exhibited similarly low feature density and minimal spatial variance, consistent with a dilute regime where particle interactions are negligible (see Figure S10 and Table S1). At 5 wt %, clear differences emerged. While both systems showed increased feature density, dispersant-treated samples maintained low spatial variance, indicating uniform particle distribution. In contrast, untreated samples exhibited significantly elevated spatial variance, reflecting the formation of particle-rich clusters and heterogeneous dispersion (Figure S10 and Table S1). These results demonstrate that dispersant effects become pronounced at higher loadings, where particle–particle interactions are significant, and quantitatively confirm that dispersant addition suppresses microsphere aggregation.

US imaging of these catheters within a phantom was conducted, and our tissue phantom exhibited similar properties to others recently reported in the literature, which supported the use of a gelatin-based phantom for our analysis.^43^ From examination of the data, it was observed that the plain TPU did exhibit some echogenicity as observed under US (Figure 7A).

**Figure 7:**
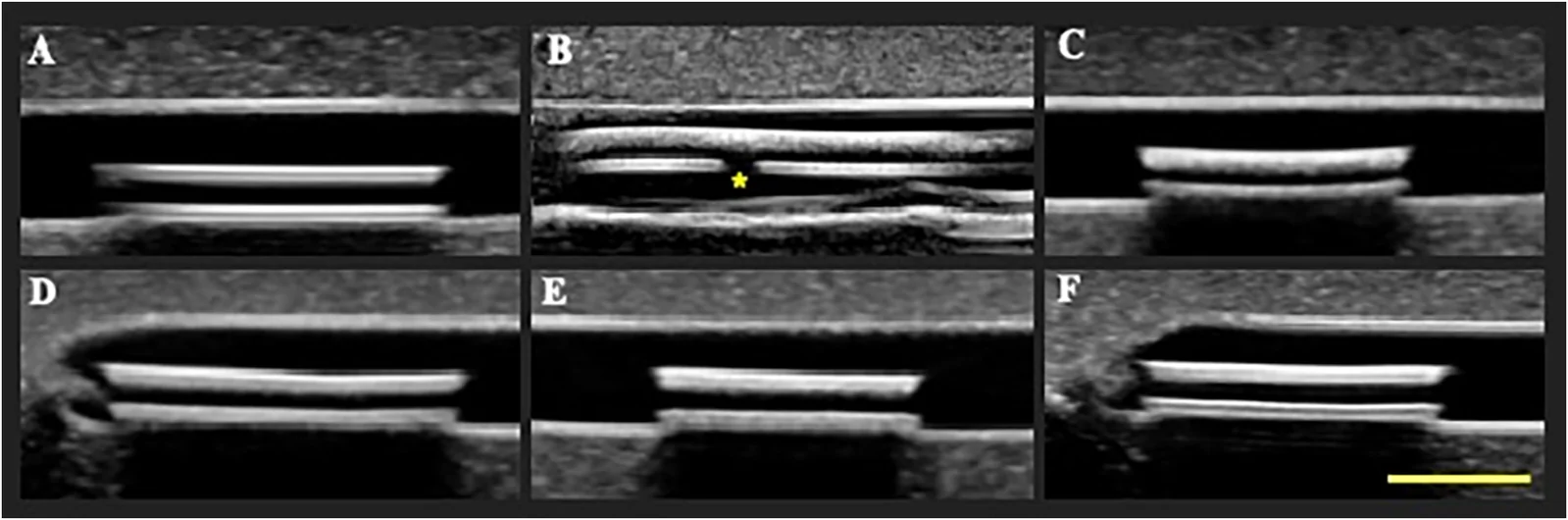
Representative sonograms of extruded tubes embedded in soft tissue phantom of (A) TPU alone, (B) with 5 wt % P40Ti MS without a coating, (C) coated with DDSD, (D) MMA2–3MPA, (E) EDGPEA, and (F)­PEGMA. Note the dark portion in the bottom tube wall (B*) is caused by agglomeration of the particles and is not an actual break in the extruded sample. Images were taken with a 12 MHz transducer at 54% gain (A, C–F) or at 84% gain (B). Scale bar represents 1 cm.

The thin white lines were indicative of a change in the relative acoustic impedance from the surrounding water to that of the polymer. It is important to note that the plain TPU also shows reflection within each wall as the double white lines of the top and bottom of the hollow tube are visible. This level of contrast would be low within the body and would require a well-trained radiologist to visualize the catheter. By addition of the higher density P40Ti MS, the catheter begins to respond to the US waves in a distinct pattern beyond the flat interfacial white line seen with the polymer alone. The sonograms of each extruded sample revealed more information about how well-dispersed the P40Ti MSs were throughout the matrix as well. In particular, while it was possible to incorporate the P40Ti MS without a compatibilizer at 5 wt % into the TPU of interest, the sonograms revealed manufacturing defects remained within the tubes. Agglomeration and dragging of the particles within the matrix became dark voids within the sonogram and increased the shadowing caused by the tube directly under these defects (Figure 7B*). After imaging, the samples were removed from the phantom and inspected for defects or air bubbles to confirm that the dark voids were attributed to processing defects and not trapped air within the tubes. The coated P40Ti MS revealed few to no such voids within the sonograms, indicating the importance of the addition of a compatibilizer. Within the DDSD and EDGPEA samples (Figure 7C,E), the texture of the glass particle loadings can still be observed in the walls of the tube. The MMA_2_-3MPA and PEG_360_MA coatings reveal a brighter and more uniform response, with more of the tube wall being visible than the plain TPU.

A signal-to-noise ratio comparison was conducted for these catheters. For all samples, a significant (*p* < 0.005) increase in SNR was noted compared to the plain TPU. The percent change in SNR was reported against the baseline SNR of the plain TPU for each of the 5 wt % loaded samples without and with coatings (Figure 8).

**Figure 8:**
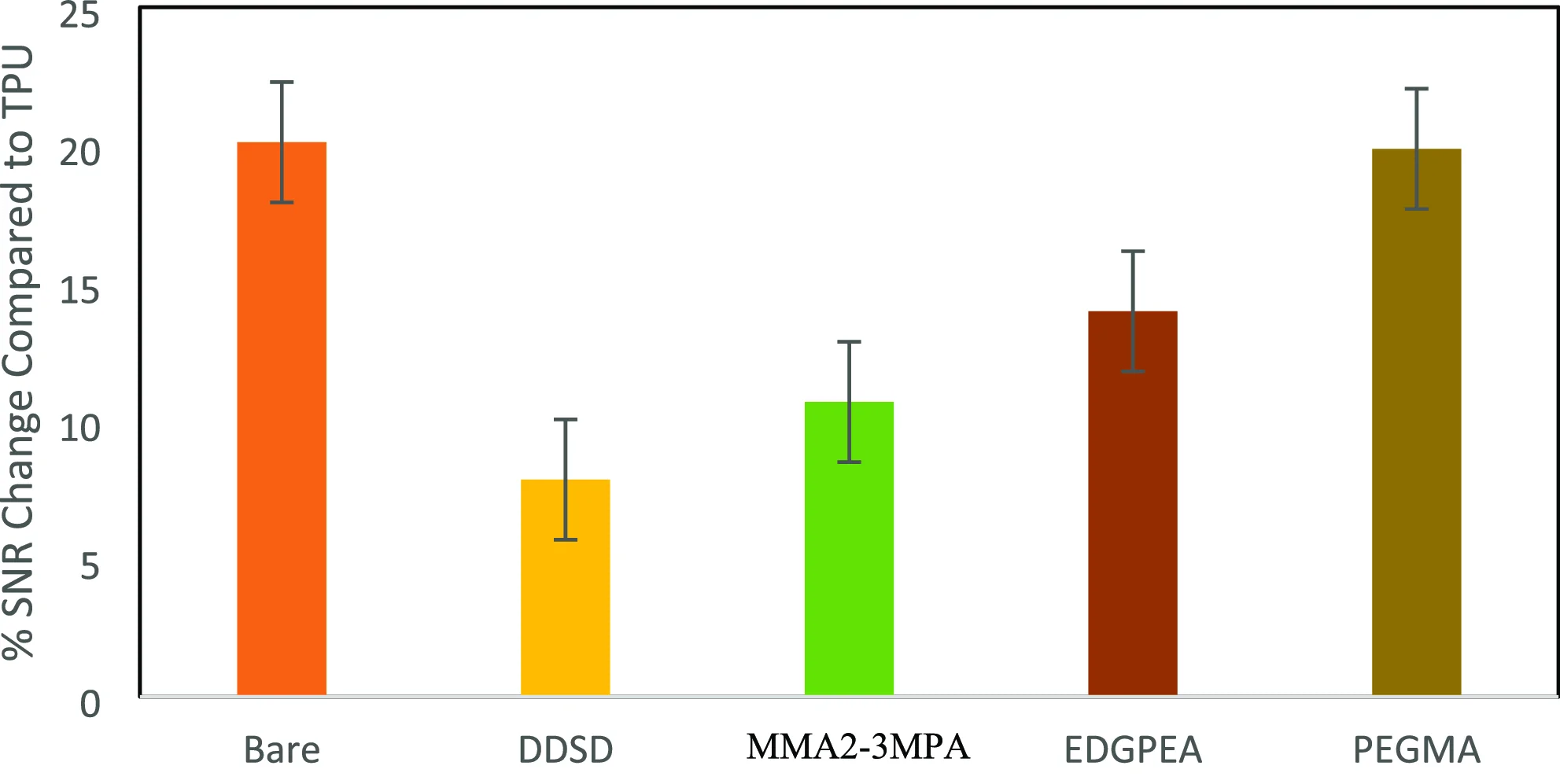
Percent increase in SNR (*n* = 5) as measured from the sonograms for each composition compared to the TPU alone. Error bars represent the standard error. All compositions displayed statistically significant (*p* < 0.005) increase in SNR over the TPU.

This was conducted because microsphere agglomeration or processing defects can increase apparent backscatter intensity and signal-to-noise ratio (SNR), but this effect does not correspond to an improved imaging performance. Aggregation produces spatially heterogeneous scatterer distributions, leading to localized high-intensity regions, increased speckle, and reduced image fidelity and reproducibility.^44,45^ In addition, clustered microspheres exhibit nonlinear and coupled acoustic responses, resulting in unpredictable signal behavior and a loss of quantitative accuracy.^46^ From a scattering perspective, increased signal in clustered systems reflects enhanced spatial correlation rather than uniform contrast and therefore does not translate to improved diagnostic imaging (Insana et al., 1990).^47^

The agglomeration noted within the bare particles (Figure 7B) led to a high SNR percent increase compared to the DDSD, MMA_2_-3MPA, and EDGPEA-coated particles (Figure 8). This has been attributed to the defects created by the poor dispersion and matrix-to-particle adhesion. PEG_360_MA displayed an SNR increase similar to that of the bare particles but without the noticeable defects observed without using a coating, suggesting that this had been achieved via good compatibilization with the matrix. The SNR that was observed appeared to increase with the level of EO character in the pendant group, which may reflect the level of hydrophobicity of these moieties influencing the level of compatibility achieved. If we compare the log *P* values of the monomers to give an indication of the characteristics of the dispersants, then the lowest SNR is from the most hydrophobic (DDSA log *P* = 4.38). Those with intermediate values (MMA = 1.38 and EDGPEA = 2.60) are slightly higher and cannot be statistically differentiated from one another, while the highest values were from PEGMA which is the most hydrophilic (log *P* = −0.8). An alternative explanation would be that the SNR is related to the steric bulk and flexibility of the pendant group. MMA and DDSA, which have the smallest and/or most flexible pendant groups, which give lower SNR values, while the longer or more bulk pendant groups in PEGMA and EDGPEA give the highest SNRs. Thus, the SNR may be affected by the amount of free volume and steric stabilization afforded by the structure around the particles being influenced by the dispersant used.

These findings align closely with broader trends observed in the field of US-responsive polymeric materials. Recent work has shown that polymer architecture, chain rigidity, and shell composition exert strong control over acoustic performance, particularly in microbubble and polymer-shelled contrast systems, where tuning chain length, segmental flexibility, and chemical composition enables precise modification of cavitation behavior, shell stability, and acoustic contrast levels.^48^ In these systems, adjusting polymer chemistry alters gas stabilization dynamics, ultrasound energy dissipation, and overall echogenic responsiveness, demonstrating a direct relationship between molecular-scale polymer design and macroscopic imaging output.^48^

This broader understanding provides a useful framework for interpreting the behavior of our dispersant-modified microsphere composites. Similar to microbubble shell optimization, the enhanced steric stabilization, tailored chain polarity, and improved polymer–polymer interactions imparted by our thiol-derived oligomeric dispersants promote superior microsphere dispersion within the TPU matrix and yield more uniform, higher-intensity ultrasound signals.

The key conclusion is that the MMA_2_-3MPA- and PEG_360_MA-coated particles reveal brighter and more uniform response, with more of the tube wall being visible than the plain TPU. This may suggest that the MMA_2_-3MPA may be more compatible with the hard segment of the TPU, while the latter has greater affinity with the soft block. These improved affinities with the TPU allowed improved dispersion of the particles to be achieved, alongside superior echogenicity being realized as a result.

## Conclusions

Our previous study documented aggregation-derived defects and limited performance at high loadings of echogenic particles. This work establishes a clear structure–property relationship between dispersant chemistry, microsphere surface modification, composite morphology, and the resulting US echogenicity of a TPU-based medical device. This was achieved by systematically comparing the use of simple small-molecule diacid dispersant (DDSD) with a family of thiol-derived oligomeric dispersants of varying polarity, hydrophobicity, monomer architecture, and chain length. It was demonstrated that the choice of dispersant exerted a significant influence on both microsphere dispersion and US echogenicity strength and uniformity and signal-to-noise ratio. The hydrocarbyl, small-molecule DDSD dispersant, provided a useful baseline to compare with both the use of (a) uncoated microspheres (previous reported data) and (b) polymeric dispersants. Its long hydrophobic tail and diacid anchoring group exhibited reliable attachment to the PBG surface and delivered measurable improvements in microsphere dispersion relative to uncoated fillers. However, the US enhancement achieved with DDSD-coated microspheres was moderate, reflecting the limited compatibility between the hydrocarbon tail and the TPU matrix. While DDSD reduced aggregation, the resulting composites still exhibited microsphere clustering at higher loadings, which in turn produced spatially heterogeneous echogenicity.

In contrast, the thiol-derived oligomeric dispersants, produced via TCT, offered further enhancement of US performance. Their acid-functional head groups anchored effectively to the oxygen-rich PBG surface, while their tunable tail chemistries enabled improved compatibility with the TPU matrix. Oligomers containing the MMA and PEGMA360 delivered the brightest and a more uniform US response. This resulted in a greater level of the tube wall being visible than the plain TPU or uncoated microspheres. DDSA and EGDPEA oligomers also performed well, yet the texture of the glass particle loadings can still be observed in the walls, making them less suitable for clinical use. The highest US signal-to-noise ratios, potentially reflecting strong steric stabilization and favorable polymer–polymer interactions were exhibited by the PEGMA. This was attributed to the oligomer offering balanced hydrophilic, PEO-like interactions with the matrix and the highest level of steric stabilization due to the pendant group size. This, in turn, supported good dispersion and so delivered strong echogenic contrast. Across all systems, the US results correlated strongly with the degree of microsphere separation observed in SEM and with the hydrophobicity shift confirmed by biphasic separation testing. The best-performing PEGMA-based dispersant exhibited and consistently delivered the highest echogenic contrast in combination with limited surface texturing. This was attributed to its ability to simultaneously providing (i) anchored strongly to the PBG surface, (ii) provided sufficient steric stabilization to prevent aggregation during melt processing, (iii) exhibited chemical compatibility with the TPU matrix, (iv) presented limited surface texturing, and (v) the highest SNR values.

Overall, this study demonstrated that tailored dispersant design guided by (a) controlled polymerization, (b) headgroup chemistry, and (c) polymer–polymer compatibility provided a powerful and scalable route to producing echogenic polymer composites suitable for real-time US tracking. By enabling reliable, high-contrast imaging without reliance on ionizing X-ray radiation, these materials offer a promising pathway toward safer and more efficient in-ward placement and monitoring of medical devices.

## Supplementary Material



## Data Availability

The data that supports the findings of this study are available from the corresponding authors upon request.

## Notes

This document provides detailed physicochemical and microstructural characterization underpinning the development of DDSD-coated P40Ti microspheres as contrast agents for enhanced ultrasound echogenicity. Scanning electron microscopy (SEM) imaging compares uncoated and DDSD-coated particles, demonstrating that the particles remain discrete and well-separated following the coating process. Thermogravimetric analysis (TGA) of the DDSD dispersant, alongside the thermal behavior of the particles, indicates that the observed weight loss is too low to be used as a definitive indicator of coating presence. In contrast, X-ray photoelectron spectroscopy (XPS), including both wide-scan and high-resolution C 1s spectra supported by detailed structural assignments of DDSD, confirms the chemical composition and carbon bonding environments, providing strong evidence for successful surface coating. Additional quantitative image analysis of particle distributions within polymer composites reveals reduced spatial variance and improved homogeneity for DDSD-coated particles compared to uncoated systems, particularly at higher loadings. These data validate the structural integrity, surface chemistry, and enhanced dispersion behavior of the coated microspheres, supporting their effectiveness as ultrasound-enhancing additives in medical device materials (PDF)
