# Supplementary material for: Contrast Agent Design to Deliver Improved Ultrasound Echogenicity from Medical Devices: A Route to In-Ward Device Placement Tracking
Source: Biomacromolecules. 2026 Jul 31;27(9):5984–96. doi: 10.1021/acs.biomac.6c00731 (PMC13580390; doi:10.1021/acs.biomac.6c00731)
Supplement: Supplementary file 1 [file bm6c00731_si_001.pdf]

Supplementary Information to support publication manuscript entitled "Contrast Agent Design to Deliver Improved Ultrasound Echogenicity from Medical Devices: A Route to In-Ward Device Placement Tracking"

Amy Stimpson,[†,‡] Jerry Contreras,[§] Bryce Roper,[§] James W. Caruana,[†] Ifty Ahmed,[‖] Cordula Hege,[†] Joseph Sefton,[†,‡] Abby R. Whittington,[*,§,⊥] and Derek J. Irvine[*,†,‡]

† Centre for Additive Manufacturing, Faculty of Engineering, University of Nottingham, University Park, Nottingham, NG7 2RD, U.K.
‡ Department of Chemical and Environmental Engineering, Faculty of Engineering, University of Nottingham, University Park, Nottingham, NG7 2RD, U.K.
§ Department of Materials Science and Engineering, Virginia Tech, Blacksburg, Virginia 24061, United States
‖ Advanced Materials Research Group, Faculty of Engineering, University of Nottingham, Nottingham, NG7 2RD, U.K.
⊥ Department of Chemical Engineering, Virginia Tech, Blacksburg, Virginia 24061, United States

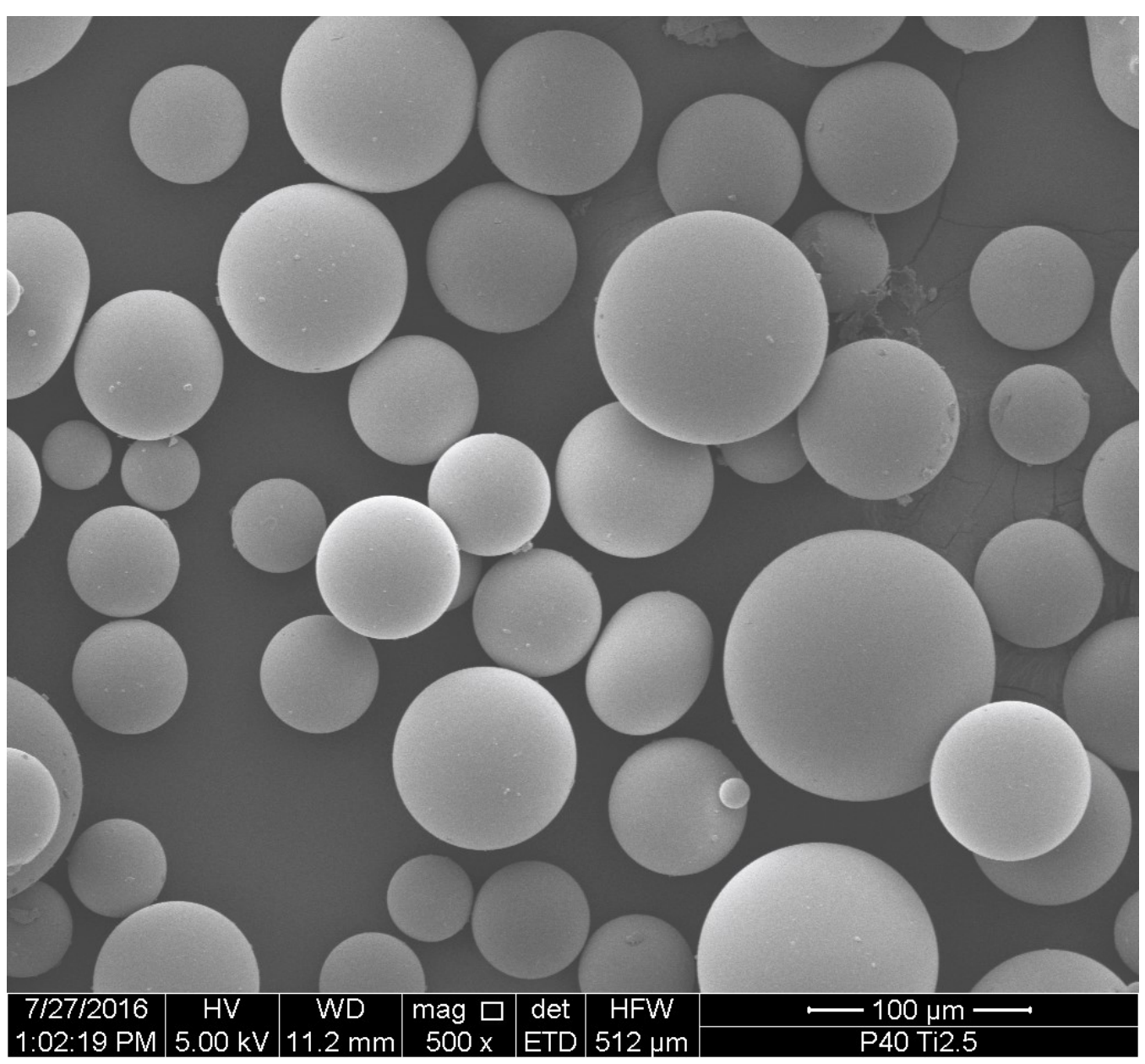

Figure S1: SEM image of uncoated P40Ti

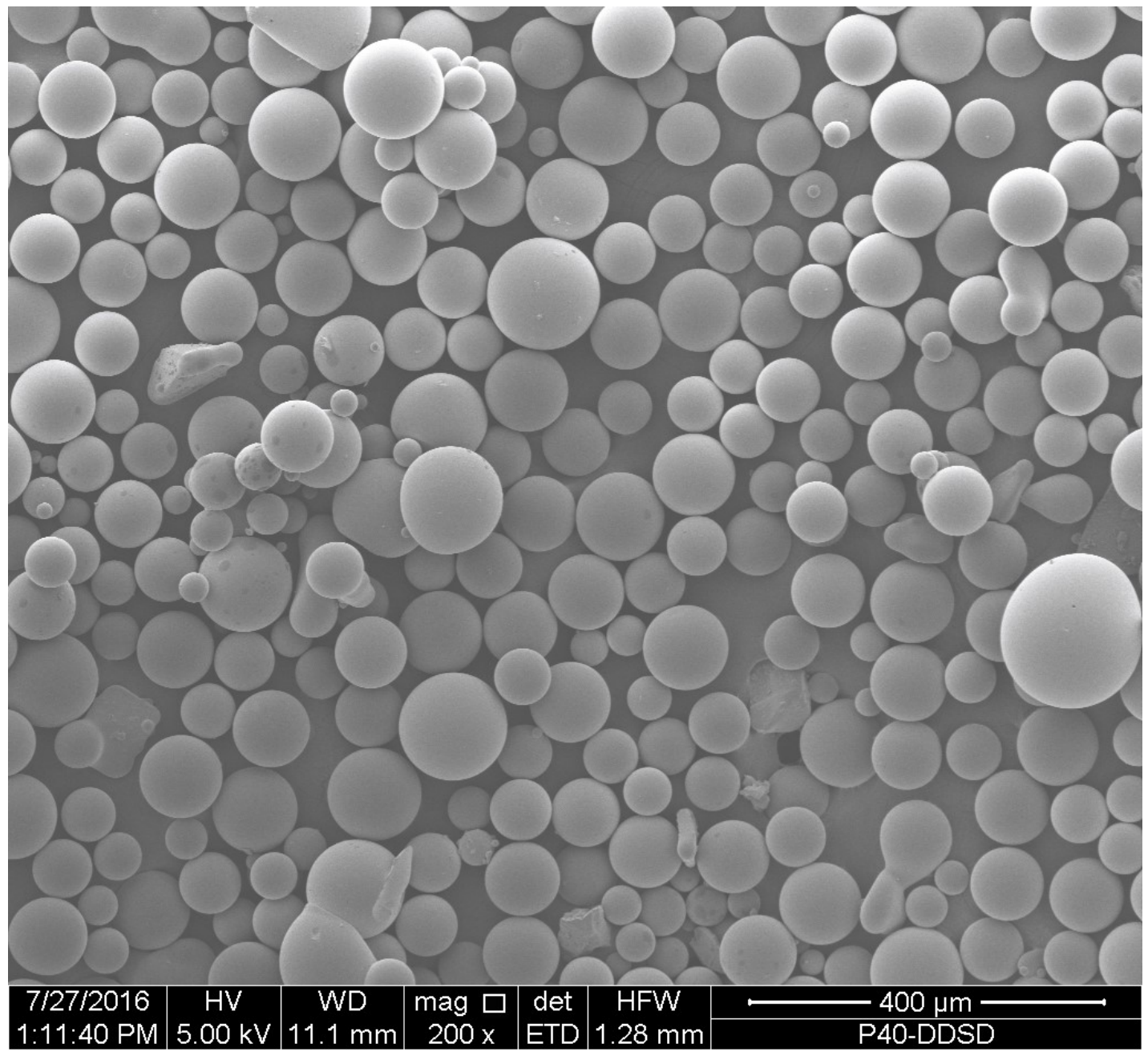

Figure S2: SEM image of P40-DDSD

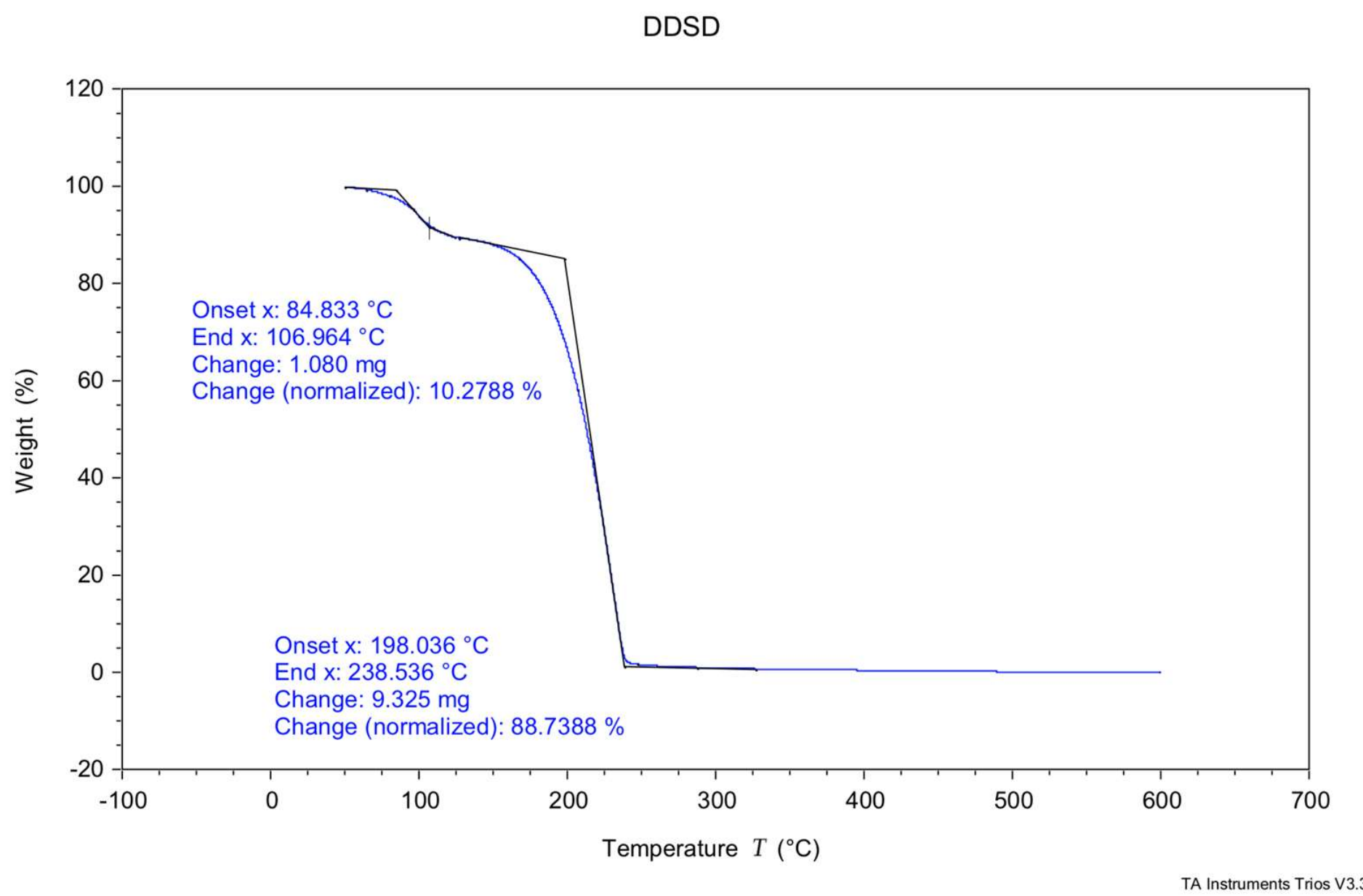

Figure S3: TGA thermogram of DDSD

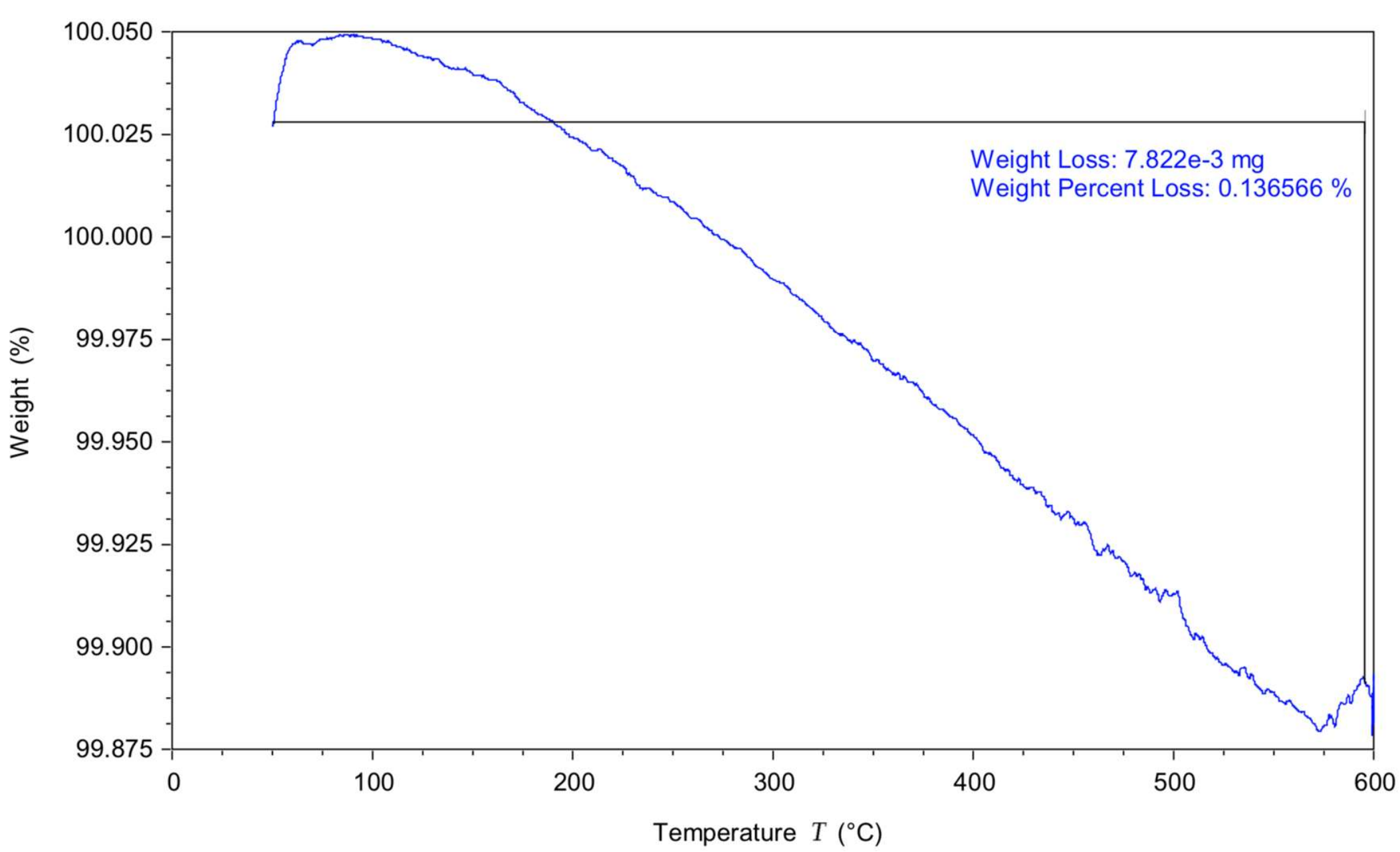

Figure S4: TGA thermogram of P40Ti microspheres

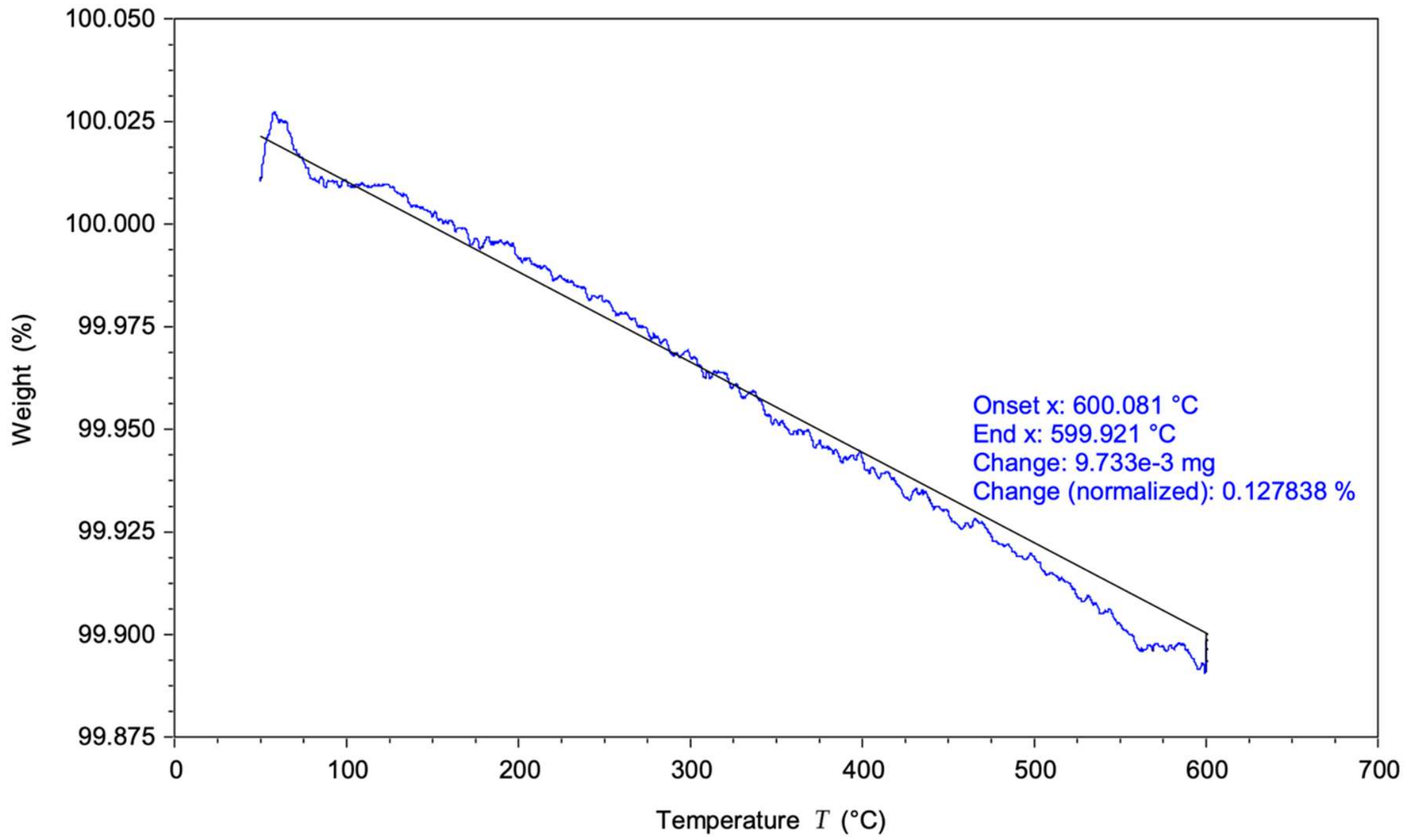

Figure S5: TGA thermogram of P40-DDSD

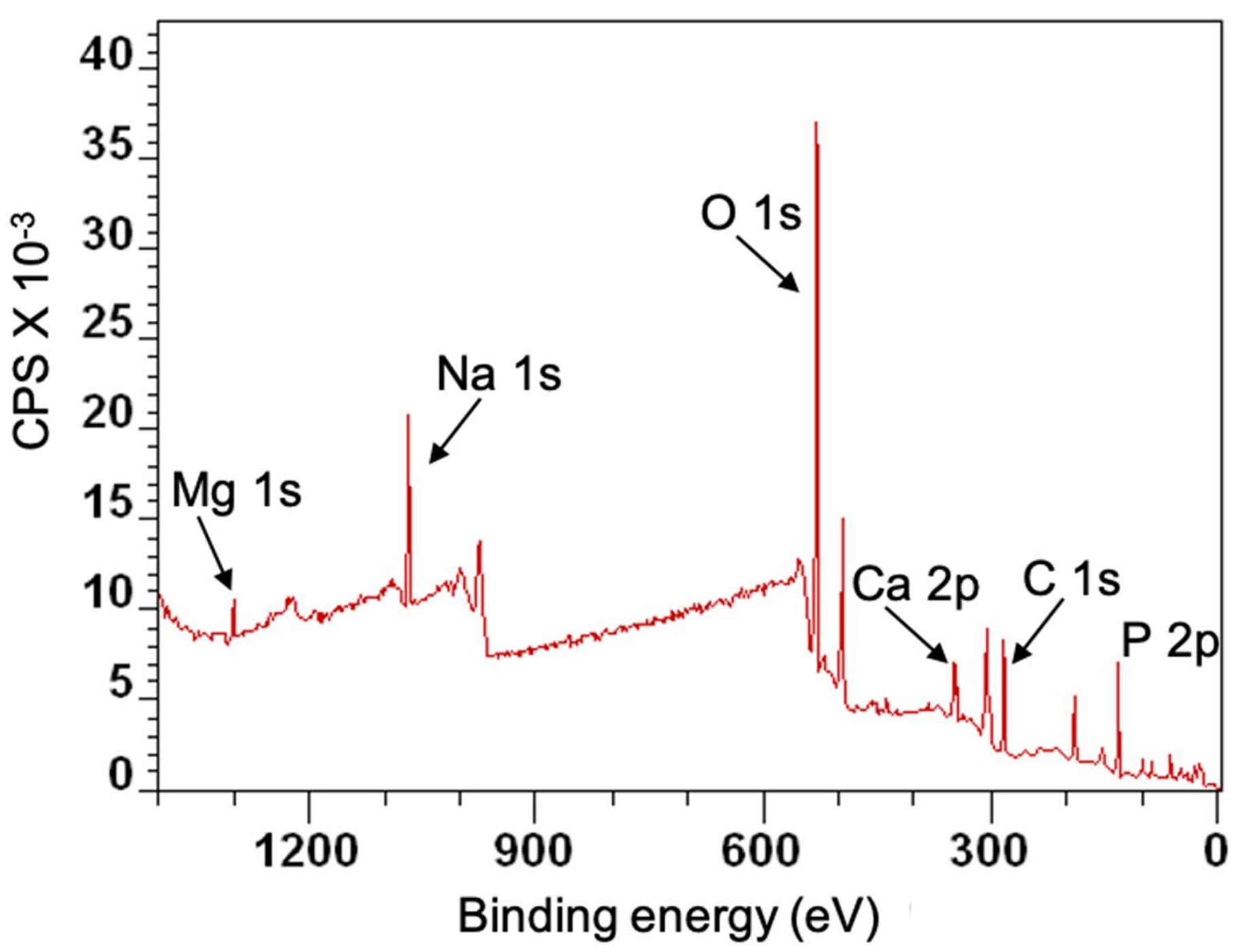

Figure S6: Example wide scan spectra of P40-DDSD with peaks assigned to elements.

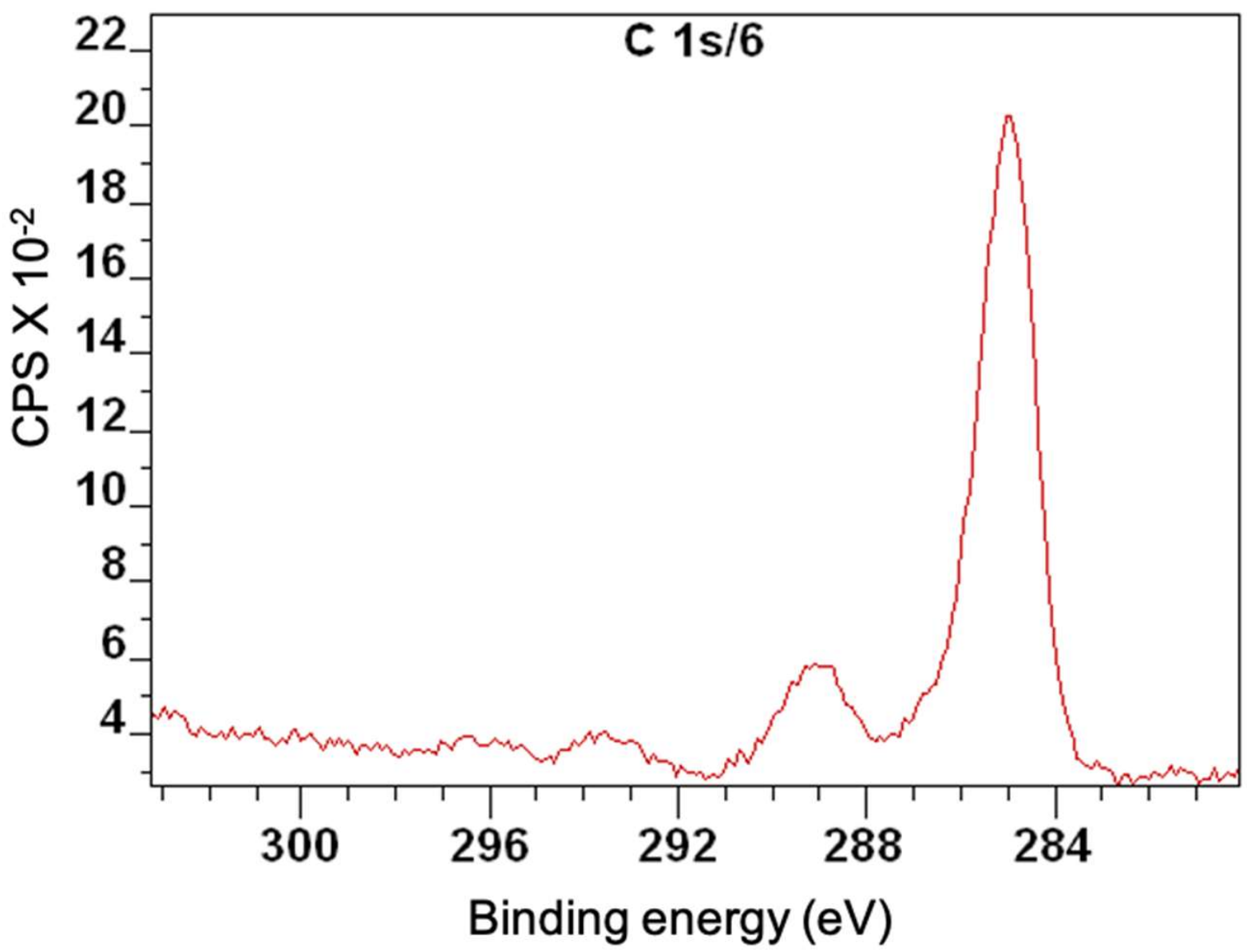

Figure S7: High-resolution C 1s scan P40-DDSD.

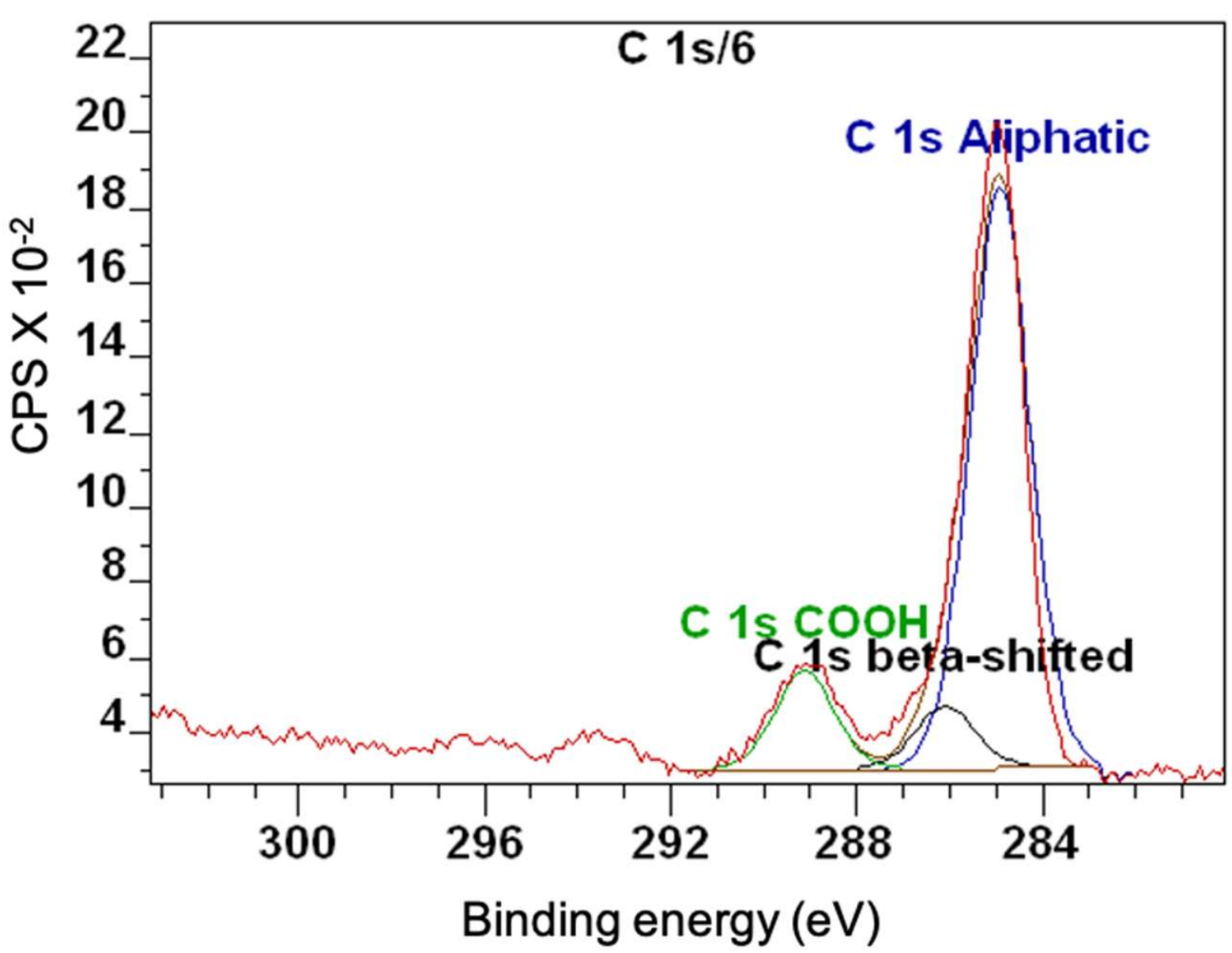

Figure S8: C 1s high-resolution scan of P40-DDSD with carbon environments fitted. Red – original spectra, blue – aliphatic component, green – COOH component, black – β-shifted component.

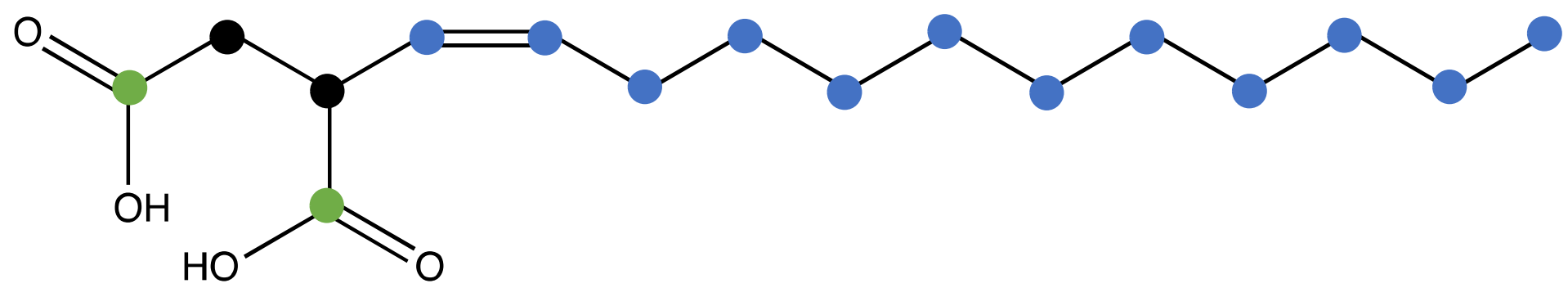

Figure S9: Structure of DDSD with carbon environments labelled in accordance to Figure S8.

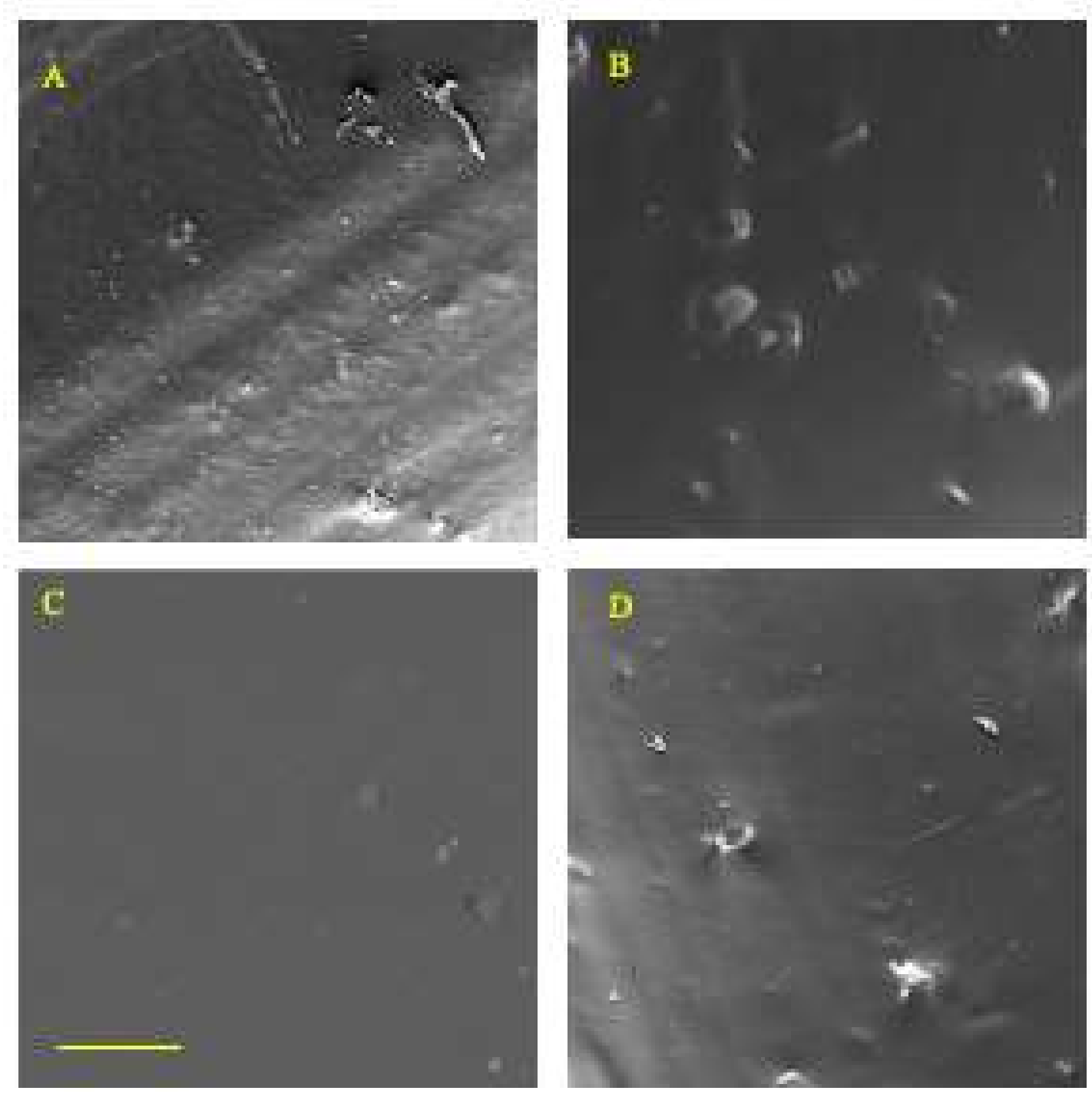

Figure S10. Semi-quantitative spatial variance analysis of representative SEM micrographs of the polymer-composite at (A) 1 wt% bare particles, (B) 5 wt% bare particles, (C) 1 wt% DDSD and (D) 5 wt% DDSD. Scale bar represents 100 **μ**m for all images.

Table S1: Compares the calculated Dark Area Fraction of each sample with baseline matrix subtracted. The Spatial Variance reveals significant (*) increase in variance for the uncoated particles compared to the coated (n = 3).

Entry	Sample Identity	Dark Area Fraction	Spatial Variance
1	1 wt% P40	0.003 ~ 0.005	Low
2	1 wt% DDSD	0.002 ~ 0.003	Low
3	5 wt% P40	0.018 ~ 0.035	Elevated*
4	5 wt% DDSD	0.008 ~ 0.015	Low / Moderate
